# Selected neuropeptide genes show genetic differentiation between Africans and non-Africans

**DOI:** 10.1186/s12863-020-0835-8

**Published:** 2020-03-14

**Authors:** Kah Yee Tai, KokSheik Wong, Farhang Aghakhanian, Ishwar S Parhar, Jasbir Dhaliwal, Qasim Ayub

**Affiliations:** 1 School of Information Technology, Monash University Malaysia, 47500 Bandar Sunway, Selangor Darul Ehsan Malaysia; 2 Monash University Malaysia Genomics Facility, 47500 Bandar Sunway, Selangor Darul Ehsan Malaysia; 3 Jeffrey Cheah School of Medicine and Health Sciences, Brain Research Institute, Monash University Malaysia, 47500 Bandar Sunway, Selangor Darul Ehsan Malaysia; 4 School of Science, Monash University Malaysia, 47500 Bandar Sunway, Selangor Darul Ehsan Malaysia

**Keywords:** Comparative genomics, Genetic variation, Neuropeptide, Population differentiation, Derived allele frequency

## Abstract

**Background:**

Publicly available genome data provides valuable information on the genetic variation patterns across different modern human populations. Neuropeptide genes are crucial to the nervous, immune, endocrine system, and physiological homeostasis as they play an essential role in communicating information in neuronal functions. It remains unclear how evolutionary forces, such as natural selection and random genetic drift, have affected neuropeptide genes among human populations. To date, there are over 100 known human neuropeptides from the over 1000 predicted peptides encoded in the genome. The purpose of this study is to analyze and explore the genetic variation in continental human populations across all known neuropeptide genes by examining highly differentiated SNPs between African and non-African populations.

**Results:**

We identified a total of 644,225 SNPs in 131 neuropeptide genes in 6 worldwide population groups from a public database. Of these, 5163 SNPs that had ΔDAF |(African - non-African)| ≥ 0.20 were identified and fully annotated. A total of 20 outlier SNPs that included 19 missense SNPs with a moderate impact and one stop lost SNP with high impact, were identified in 16 neuropeptide genes. Our results indicate that an overall strong population differentiation was observed in the non-African populations that had a higher derived allele frequency for 15/20 of those SNPs. Highly differentiated SNPs in four genes were particularly striking: *NPPA (rs5065)* with high impact stop lost variant; *CHGB (rs6085324, rs236150, rs236152, rs742710 and rs742711)* with multiple moderate impact missense variants; *IGF2 (rs10770125)* and *INS (rs3842753)* with moderate impact missense variants that are in linkage disequilibrium. Phenotype and disease associations of these differentiated SNPs indicated their association with hypertension and diabetes and highlighted the pleiotropic effects of these neuropeptides and their role in maintaining physiological homeostasis in humans.

**Conclusions:**

We compiled a list of 131 human neuropeptide genes from multiple databases and literature survey. We detect significant population differentiation in the derived allele frequencies of variants in several neuropeptide genes in African and non-African populations. The results highlights SNPs in these genes that may also contribute to population disparities in prevalence of diseases such as hypertension and diabetes.

## Background

Neuropeptide genes [1, 2] are no different when it comes to genetic risk. As their name indicates, these genes code for neuropeptides are peptide molecules that are synthesized and released from nerve cells in the brain and act either at the local level in the brain or affect distant organs. For more than 30 years following the discovery of the first neuropeptide by Van Euler in 1931, studies [3] were geared towards the role of these peptides as signaling molecules in the peripheral and central nervous systems, where they act as fine tuners of neurotransmissions that control the balance between neuronal inhibition and excitation. A large number of neuropeptides were identified during this period [4, 5].

Neuropeptides are also expressed in the endocrine and immune system and play a major role in physiological homeostasis. They intersect the immune, nervous and endocrine systems through autocrine, neurocrine, paracrine and endocrine manners, thus playing a core role in influencing postsynaptic cells in a large target area [6]. In physiological homeostasis, neuropeptides act as peptide hormones regulating functions such as feeding behavior, reproduction, stress response, energy homeostasis, cognition, pain and blood pressure. Additionally, they perform their physiological processes by binding to corresponding receptors [7] and an abundance of neuropeptides has been reported in almost every system of the human body [4, 6, 8]. To date, from over 1000 predicted peptides encoded in the human genome, there are now over 100 known neuropeptide genes in the human and undoubtedly many more that are yet to be identified and annotated [9].

As humans migrated into new frontiers outside Africa their populations became fragmented and genetically differentiated. This genetic diversity can also be a source of differences in genetic risk for particular ailments between different populations. For example, variant *rs2478523* in the *AGT* gene shows an increase in the risk of high altitude polycythemia (HAPC) in the Tibetan population while in the Han population, *rs699*, *rs4762* and *rs5051* are associated with reduced HAPC susceptibility [10]. Also, in the USA population, a minor allele *rs5065* in *NPPA* was identified as a marker of increased cardiovascular risk [11], and in the North Indian population, *rs1042571* in *POMC* was shown to increase the risk of obesity [12]. Due to the importance of neuropeptides, even minor variations in neuropeptide genetic structure can lead to vastly different physiological effects. Differences in neuropeptide genetics can thus serve as better markers or indicators for the susceptibility of a specific population for certain diseases, aiding in population health measures. Even so, the knowledge available on the variability and expression pattern of these neuropeptide genes in different modern human populations is limited at the moment. The majority of the studies conducted on these genes [13–15] so far have tended to focus on one specific neuropeptide in one specific population [11, 16–19].

The rapid development in sequencing technology and decreasing costs of genome sequencing now proffer an unbiased examination of human genetic variation and have led to the development of several large scale human whole exome and whole genome databases, such as the 1000 Genomes Project [20], the Trans-Omics for Precision Medicine (TOPMed) and the Genome Aggregation Database Consortium [21], that aim to translate these gains into clinical medical practice based on personalized genomics. The major goal of these projects is to establish a comprehensive catalogue of all detectable variations, which is essential for characterizing human genetic diversity as well as identifying risk variants associated with human diseases. By being able to monitor the variations in multiple genes simultaneously in a particular population and forming a genomic profile, it is possible to deduce their influence on a disease, or even overall health.

In this study, we analyzed the genetic variation in continental human populations across known neuropeptide genes. In particular, we examined single nucleotide polymorphisms (SNP) that were highly differentiated between African and non-African populations in publicly available datasets, to gain insights about the patterns of genetic variations in genes that code for neuropeptides and examine whether any are undergoing any adaptive selection in these populations.

## Results

### Human neuropeptide genes

**Fig. 1:**
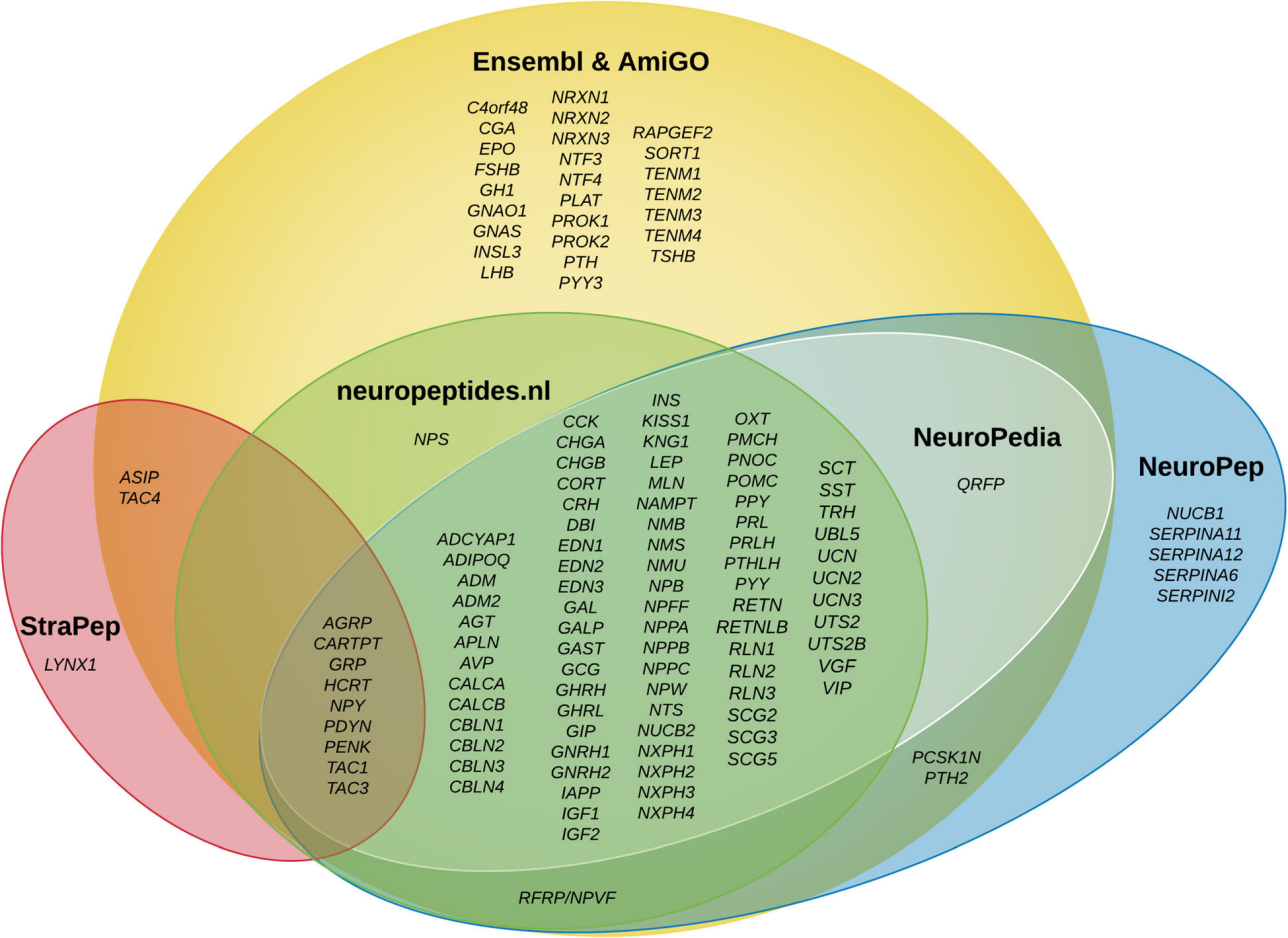
Human neuropeptide genes obtained from the online database and literature survey

A total of 105 neuropeptide genes were identified from four neuropeptide databases; StraPep [22], neuropeptides.nl [23], NeuroPedia [24] and NeuroPep [25], by using the search term “*Homo sapiens*”. An additional 26 neuropeptide genes were identified by Ensembl [26] and AmiGO Gene Ontology [27]. Therefore, the final list comprised of 131 human neuropeptide genes (Fig. 1, Additional file 1: Table S1).

### Variation in neuropeptide genes

Using the whole genome sequence data, we extracted variants for the 131 neuropeptide genes in 15,164 individuals belonging to 6 different populations, Africans, Latino, Ashkenazi Jewish, East Asian, Finnish and Non-Finnish Europeans. A total of 769,597 variable sites were identified in the 131 neuropeptide genes (Additional file 2: Table S2). We filtered out 125,372 indels variants and retained a total number of 644,225 SNPs for downstream analysis because ancestral alleles could not be obtained for the indels.

### Highly differentiated SNPs in Africans and non-Africans

**Fig. 2:**
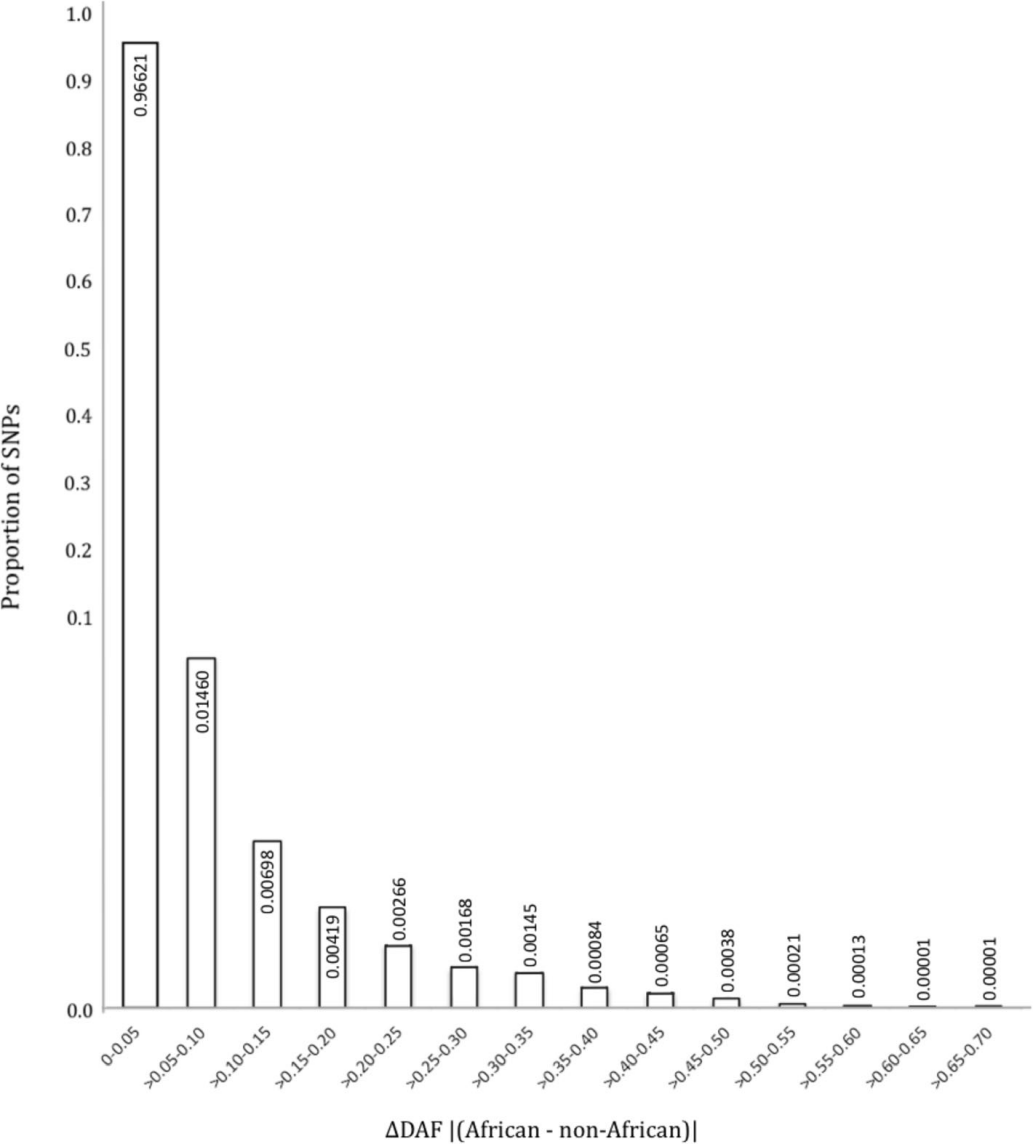
Proportion of SNPs binned on basis of the absolute differences in African and non-African derived allele frequencies (ΔDAF). Numbers inside and outside the bars refer to the proportion of SNPs that were observed in each bin

**Fig. 3:**
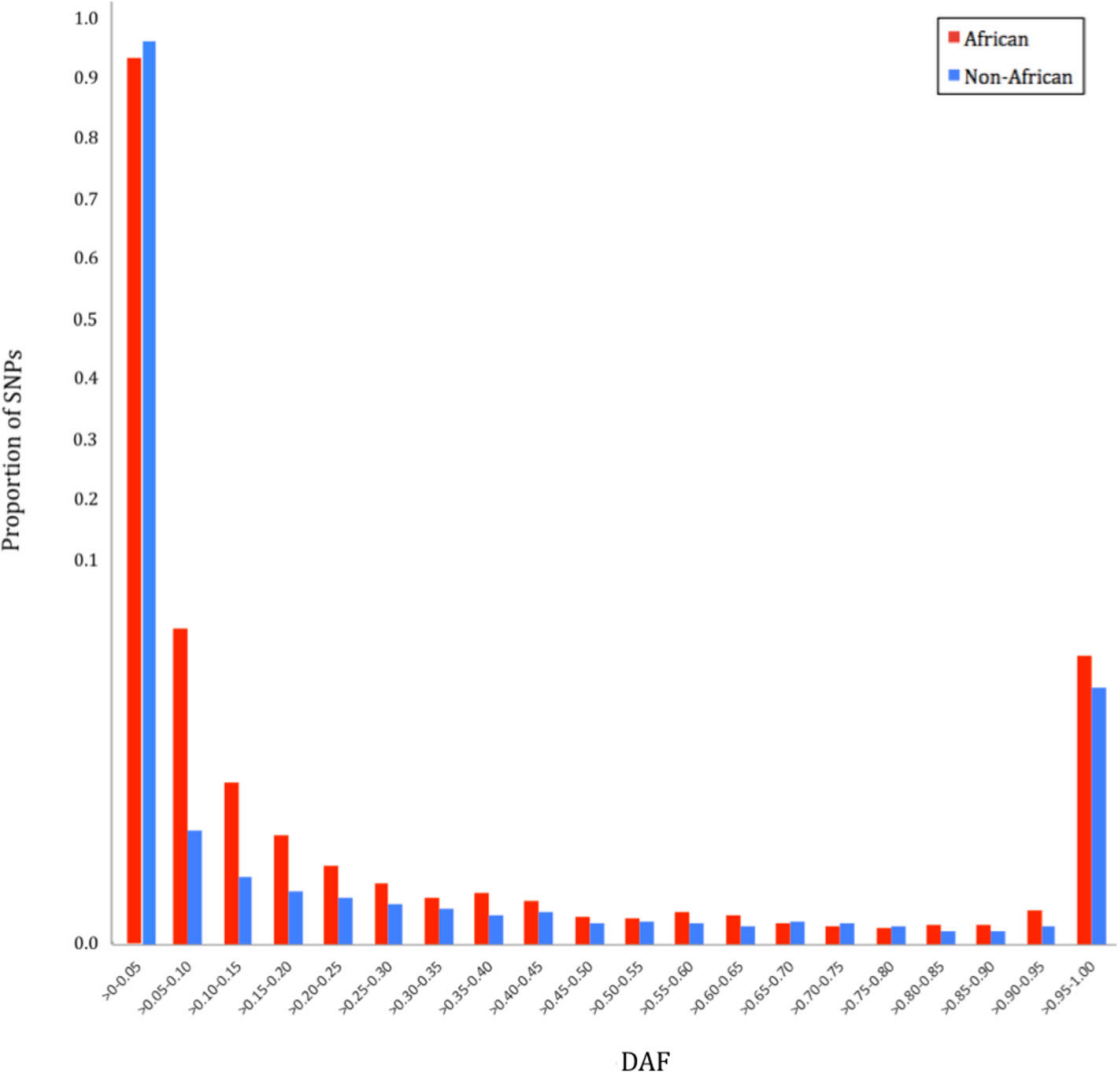
Proportion of neuropeptide gene SNPs in various derived allele frequency bins in African and non-African populations

SNPs in neuropeptide genes, that had absolute differences in derived allele frequencies (DAF) between African and non-African populations equal to or more than 0.20, were identified and functionally annotated (Figs. 2 and 3). A cutoff point of DAF ≥ 0.20 was selected because it represented the extreme (< 1%) outliers amongst the 644,225 SNPs (Additional file 3: Figure S1). Overall, 5163 of 644,225 SNPs met this criteria (Additional file 4: Table S3). Ensembl Variant Effect Predictor (VEP) tool was used to annotate these 5163 SNPs to identify missense variants or SNPs with high impact functional consequences (Additional file 5: Figure S2). A total of 20 SNPs (Table 1), that included 19 moderate impact missense SNPs and one high impact loss of stop codon, were identified in 16 different neuropeptide genes. An overall strong population differentiation was observed in the non-African populations that had a higher derived allele frequency for 15/20 of these SNPs.

**Table 1:** Twenty SNPs in neuropeptide genes that are highly differentiated between Africans and non-Africans

SNP ID	Chromosome	HGNC Symbol	Ref	Ancestor	Derived	ΔDAF |(African - non-African)|	Feature key^a^ (Position)	F_ST_ YRI vs CEU	F_ST_ YRI vs CHB	eQTL (↓decrease; ↑increase)
*rs3842753*	11	*INS*	T	T	G	0.50		0.44	0.70	*TH↑; IGF2-AS↓; IGF2↑*
*rs3847193*	9	*QRFP*	G	A	G	0.42	Propeptide (19–90)	0.34	0.68	*QRFP↓; FIBCD1↑*
*rs2291725*	17	*GIP*	T	T	C	0.38	Propeptide (95–153)	0.49	0.64	*AC091133.1↓; ATP5G1↑; ATP5G1↓; CDK5RAP3↑; HOXB-AS1↑; HOXB2↑; SNF8↑; SUMO2P17↑; TTLL6↓; UBE2Z↑; UBE2Z↓*
*rs699*	1	*AGT*	A	G	A	0.36		0.46	0.07	*AGT↑; AGT↓; COG2↑*
*rs6788319*	3	*UTS2B*	C	T	A	0.34	Signal peptide (1–28)	0.27	0.22	*UTS2B↓; UTS2B↑; CCDC50↓; OSTN-AS1↑*
*rs10770125*	11	*IGF2*	A	A	G	0.32		0.30	0.21	*IGF2-AS↓*
*rs5065*	1	*NPPA*	A	G	A	0.30		0.16	0.42	*CLCN6↑; MTHFR↑; NPPA↑; NPPA-AS1↑*
*rs2286472*	16	*NPW*	A	C	A	0.29	Propeptide (65–165)	0.17	0.18	*NPW↓; RP11-304 L19.13↑; SLC9A3R2↑; SYNGR3↑*
*rs1885137*	14	*SERPINA11*	T	T	G	0.28		0.36	0.09	*RP11-349I1.2↓; SERPINA11↓*
*rs7121*	20	*GNAS*	C	T	C	0.28		0.35	0.12	*GNAS↑; CTSZ↑*
*rs757081*	11	*NUCB2*	C	C	G	0.26		0.39	0.40	*RP1-239B22.5↓; PIK3C2A↓; OR7E14P↓; NUCB2↓; NCR3LG1↓; KCNJ11↓*
*rs990310*	10	*NPS*	C	C	T	0.24	Signal peptide (1–23)	0.30	0.32	
*rs742711*	20	*CHGB*	G	G	A	0.21		0.13	0.19	*KANK1P1↑; CHGB↑*
*rs236152*	20	*CHGB*	C	C	G	0.21		0.13	0.02	*CHGB↑; KANK1P1↑; LRRN4↓; MCM8↓; RP5-1056H1.2↑*
*rs6085324*	20	*CHGB*	T	T	A	0.21		0.13	0.19	*KANK1P1↑; CHGB↑;*
*rs1799917*	16	*GNAO1*	A	G	A	0.21		0.10	0.28	*AMFR↑; AMFR↓; BBS2↑; RP11-413H22.3↓*
*rs236150*	20	*CHGB*	G	G	C	0.21	O-glycosylated (116–120)	0.22	0.22	
*rs34123523*	16	*AGRP*	C	C	T	0.21	Propeptide (21–82)	0.16	0.21	
*rs1058046*	17	*PYY*	G	G	C	0.20	Propeptide (68–97)	0.11	0.05	*PYY↓*
*rs742710*	20	*CHGB*	C	C	T	0.20		0.21	0.02	

^a^*Feature key refer to the Post-translational modifications (PTM) events in the protein and position refer to the PTM events position in the protein*

### Genes of interest

Twenty SNPs that were highly differentiated (ΔDAF ≥0.20) between Africans and non-Africans occurred in 16 of 131 neuropeptide genes. Their functional consequences were analyzed using available phenotype data in Genome Wide Association Studies (GWAS) catalogue [28], Online Mendelian Inheritance in Man (OMIM) [29] and gene expression data from Genotype-Tissue Expression (GTEx) portal [30] (Table 1). Median-joining haplotype networks were constructed for these SNPs to investigate the relationship between the African and non-African haplotypes (Additional file 6: Figure S3). To compare how unusual these haplotype networks were we also generated networks for genomic regions where no SNPs had ΔDAF ≥0.20. (Additional file 7: Figure S4). As expected there were no high frequency population specific haplotypes.

Variants in four of these genes (*NPPA, CHGB*, *IGF2* and *INS*) were especially striking because of the following salient features: *NPPA* with a high impact stop lost variant (*rs5065)*; *CHGB* with multiple moderate impact missense variants *(rs6085324, rs236150, rs236152, rs742710 and rs742711)*; *IGF2 (rs10770125)* and *INS (rs3842753)* with moderate impact missense variants that are in linkage disequilibrium. These variants are further examined in the following sections.

#### *NPPA*

**Fig. 4:**
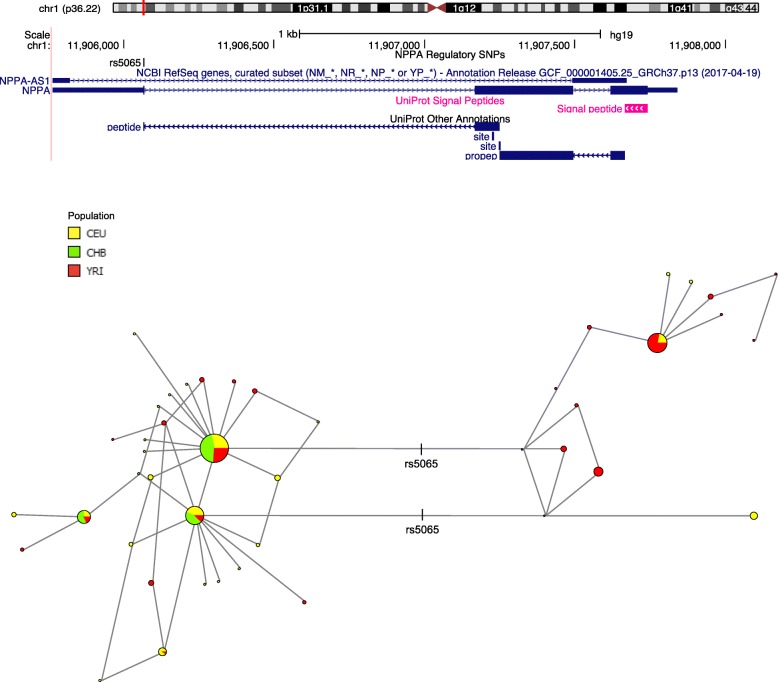
NCBI RefSeq and UniProt annotations track with haplotype network showing a 2 kb region encompassing *NPPA* in Africans (YRI), East Asians (CHB) and Europeans (CEU)

The SNP (*rs5065*) in *NPPA* has been associated with cardiovascular disease risk [11, 31] and acute coronary syndrome [32]. The derived allele frequency is significantly higher in non-Africans (88%) as opposed to Africans (59%). A haplotype network based upon 94 SNPs in a 2 kb genomic region encompassing *NPPA* (Fig. 4) clearly shows *rs5065* on the branch separating two main haplotypes, one comprising mostly of African haplotypes with frequency of 0.20 and the other including all continental groups with frequency of 0.72.

#### *CHGB*

Five highly differentiated SNPs occurred in the *CHGB* gene *(rs6085324, rs236150, rs236152, rs742710* and *rs742711)*. Three of these SNPs *(rs236150, rs236152 and rs742710)* had a high derived allele frequency in African populations and two SNPs (*rs6085324* and *rs742711)* in non-Africans (Table 2). All five SNPs were associated with stress that arises due to changes in blood pressure in Southern Californians, including sub-Saharan African and European ancestry groups [33]. Moreover, two SNPs (*rs6085324* and *rs742711*) have been associated with schizophrenia in the Korean population [19] and SNP *rs236152* has also been associated with schizophrenia in the Japanese population [16].

**Table 2:** Derived allele frequencies of highly differentiated SNPs in *CHGB* in Africans and non-Africans

SNP ID	African Derived Allele Frequency	non-African Derived Allele Frequency	ΔDAF |(African - non-African)|
*rs6085324*	0.1075	0.3187	0.2112
*rs236150*	0.2102	0.0019	0.2083
*rs236152*	0.6309	0.4176	0.2133
*rs742710*	0.2513	0.0506	0.2007
*rs742711*	0.1045	0.3184	0.2140

**Fig. 5:**
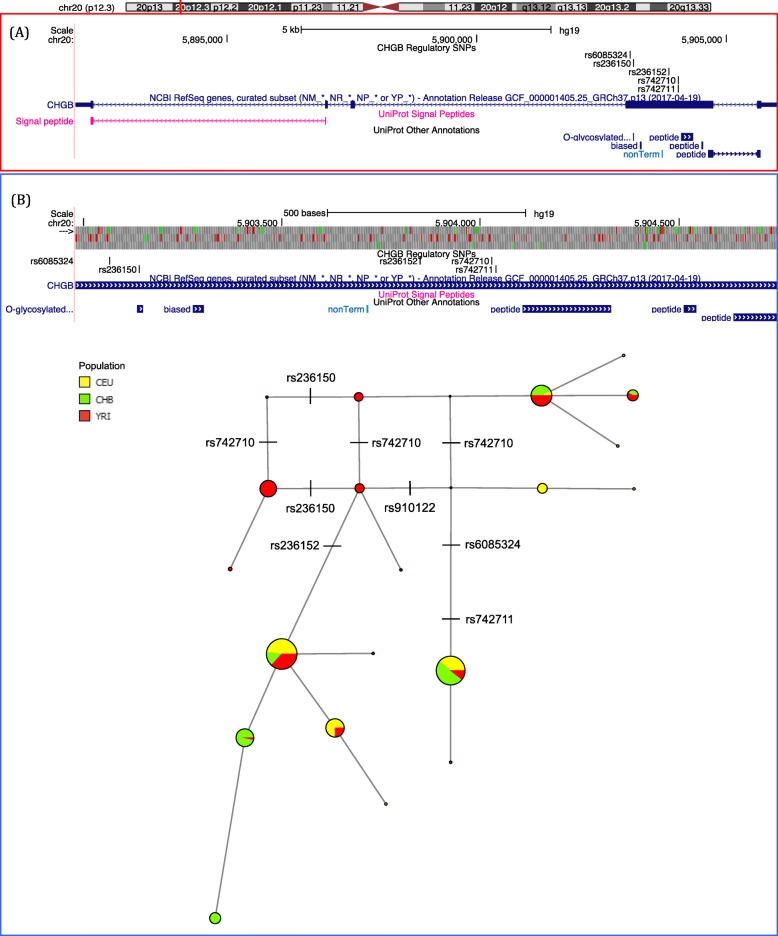
Genomic region and haplotypes for *CHGB*. **a** NCBI RefSeq and UniProt annotations track for a 14 kb genomic region on chromosome 20 encompassing the *CHGB* gene. The location of the five highly differentiated SNPs is shown above the exon (blue box). **b** NCBI RefSeq and UniProt annotations track with haplotype network of a 1 kb exonic region containing the highly differentiated SNPs within *CHGB* exon 4 in Africans (YRI), East Asians (CHB) and Europeans (CEU)

The relationship between these 5 SNPs was further explored by using Africans and non-Africans allele linkage disequilibrium (LD) (Table 3). A median-joining haplotype network was constructed using 1000 Genomes Project continental populations representing Africans, East Asians and Europeans [20]. Two haplotype networks were constructed, one consisting of 411 SNPs from the whole *CHGB* 14 kb genomic region (Additional file 8: Figure S5) and another comprising of 57 SNPs (including the 5 highly differentiated variants) in a 1 kb region of *CHGB* exon 4 (Fig. 5a-b). The haplotype network shows that four SNPs, including three of the five highly differentiated ones (*rs236152, rs6085324* and *rs742711*), separate the two major haplotypes, whereas the remaining 2 SNPs (*rs236150* and *rs742710*) mainly separate other Africans and minor non-Africans haplotypes from one another.

**Table 3:** Pairwise linkage disequilibrium (LD) between five highly differentiated *CHGB* SNPs in Africans (above diagonal) and non-Africans (below diagonal)

SNP ID	*rs6085324*	*rs236150*	*rs236152*	*rs742710*	*rs742711*
*rs6085324*		0.022	0.040	0.030	1
*rs236150*	0.020		0.156	0.102	0.022
*rs236152*	0.502	0.054		0.214	0.040
*rs742710*	0.057	0.006	0.153		0.030
*rs742711*	1	0.020	0.502	0.057

#### *IGF2 and INS*

The SNP (*rs10770125*) in *IGF2* and (*rs3842753*) in *INS* are located close together on chromosome 11. GTEx data shows that *IGF2* is highly expressed in the Adipose – Visceral (Omentum) and *INS* is highly expressed in the pancreas. The derived allele frequencies of both SNPs are higher in non-Africans as compared to Africans (Table 4). The relationship between these 2 SNPs was further studied using LD and haplotype network. The result show a higher LD in Africans (r^2^ = 0.336) than in non-Africans (r^2^ = 0.056). A haplotype network based upon 65 SNPs in a 1 kb genomic region of *IGF2* (Additional file 9: Figure S6) and a haplotype network based upon 66 SNPs in a 1 kb genomic region of *INS* (Additional file 10: Figure S7) were constructed. As expected, in both networks non-Africans exhibit high frequency haplotypes that have derived alleles for both these SNPs. A study [34] linked *rs3842753* to improved identification of atypical Type 2 Diabetes (T2D) patients in the Uruguayan population of predominantly European ancestry. In a separate study of European American descents in the GoKinD project [35], *IGF2 rs10770125* has been associated with diabetic nephropathy in male patients with T1D, but not in female patients [36].

**Table 4:** Derived allele frequencies and ΔDAF for highly differentiated SNPs in *IGF2* and *INS* in Africans and non-Africans

SNP ID	African Derived Allele Frequency	non-African Derived Allele Frequency	ΔDAF |(African - non-African)|
*rs10770125*	0.1750	0.4979	0.3229
*rs3842753*	0.2568	0.7581	0.5013

## Discussion

We used genome sequence data from six different populations groups in the Genome Aggregation Database (gnomAD) to extract variants for 131 neuropeptide genes. Using differences in derived allele frequencies we identified 20 highly differentiated SNPs between Africans and non-African populations in 16 neuropeptide genes (Table 1). Functional analysis of these highlighted the pleiotropic effects of these neuropeptide genes and their association with complex diseases such as hypertension and diabetes, the prevalence of which is known to differ between individuals of African and European ancestry [33, 34].

The high impact stop lost variant *(rs5065)* in *NPPA* has been associated with increased acute coronary syndrome [32] and cardiovascular risk [11, 31]. *NPPA* encodes a protein implicated in the control of extracellular fluid volume and electrolyte homeostasis and is highly expressed by the heart muscle. Furthermore, the ventricular expression of this gene is strongly increased in the cardiac muscle cells of the mice during stress [37].

A number of these highly differentiated SNPs were in genes that help regulate the amount of intracellular calcium that is known to play a crucial role in the regulation of cardiovascular functions. An increase in calcium in vascular smooth muscle cells leads to an augmented muscular tone which further increases vascular resistance that eventually raises the blood pressure [38]. One such gene is *CHGB* [39] that stimulates catecholamine secretion [40]. Common genetic variation at the *CHGB* locus, especially in the proximal promoter, influences *CHGB* expression, catecholamine secretion and the early heritable responses to environmental stress and is associated with changes in blood pressure in the sub-Saharan African and European ancestry groups [33]. Five missense variants *(rs6085324, rs236150, rs236152, rs742710* and *rs742711)* that lie in a single exon have high ΔDAF between Africans and non-Africans and three *(rs236152, rs742711* and *rs6085324)* of these (Table 1) are associated with increased *CHGB* expression in the GTEx dataset [30]. Of these three SNPs one *(rs236152)* has a higher derived allele frequency (63%) in Africans. Another close SNP *(rs236150)* that also has a higher derived allele frequency (21%) in Africans is also predicted to be differentially O-glycosylated. Another calcium binding protein with a highly differentiated SNP *(rs757081)* was *NUCB2*. *NUCB2* shares a 60% sequence homology with *NUCB1* in the human and mouse genome [41] and plays an important role in homeostatic functions associated with stress response [42], where its expression increased intracellular calcium concentration by protein kinase C activation in cultured rat cultured rat dorsal root ganglion neurons [43]. This SNP has also been associated with systolic blood pressure, mean arterial pressure and pulse pressure in individuals with European ancestry [44], and in African Americans it has been associated with both systolic and diastolic blood pressure [45]. *AGT*, another gene with a highly differentiated SNP, *rs699*, with a derived allele frequency of 17% in Africans, has also been associated with hypertension in African populations [46]. Based on the single-tissue eQTL in GTEx, the *NUCB2 rs757081* and *AGT rs699* decreases their gene expression levels in several tissues and both SNPs have been associated with hypertension in the GWAS catalogue [28].

Several of the other genes, including *INS*, *GIP* and *IGF2* with highly differentiated SNPs are involved in regulating glucose homeostasis. Evidence from epidemiological studies suggests that African Americans are also more insulin resistant and have higher insulin responses to glucose than European Americans [47]. The balance between insulin and glucagon levels is crucial in maintaining glucose homeostasis [48]. *INS rs3842753* with a derived allele frequency of more than 75% in non-African populations has been identified as a marker for atypical T2D in the Uruguayan population [34], while *IGF2 rs10770125* has been associated with diabetic nephropathy in people with European American ancestry [36]. *GIP* is secreted from K cells and acts on pancreatic beta cells to stimulate the release of insulin. Using the HGDP-CEPH project and the Human Genome Center at the University of Tokyo datasets, a previous study [49] showed that the derived frequency of *rs2291725* is significantly higher (> 60%) in the majority of East Asian populations while varying widely in other populations, ranging between 0.0–9.5% in sub-Saharan Africans and increasing to > 40% in European and Middle Eastern populations. We also noted a low derived allele frequency of 14% for this SNP in the Africans and a significantly higher derived allele frequency of 52% for non-Africans. The highest derived allele frequency was also seen in East Asian populations with frequency of 0.75. *NUCB2 rs757081* variant was also associated with the decreased risk of developing T2D in Chinese Han population [50]. The *CHGB* gene is also essential for adequate secretion of islet hormones in mice, where its deficiency led to a phenotype with some hallmarks of human T2D including loss of initial rapid insulin secretion [51]. Three missense variants *(rs6085324, rs742711 and rs236152)* have been associated with schizophrenia and increased risk for T2D [19].

A major limitation of the study was the non-availability of individual sequences in the gnomAD dataset. Therefore, selected sequences from the 1000 Genomes Project continental populations representing Africans and non-Africans were used to construct the haplotype networks, compute LD and F_ST_ for the highly differentiated SNPs. As expected haplotype networks for the highly differentiated genes show population sub-structure with high frequency population specific haplotypes. However, this could not be considered an unusual feature, because it is dependent upon the underlying linkage disequilibrium between SNPs in these populations and confounded by selection and demography.

## Conclusions

Our study shows substantial population differentiation between African and non-African, as measured by differences in derived allele frequencies, in variants located in 131 neuropeptide genes. Twenty outlier SNPs with ΔDAF |(African – non-African)| ≥ 0.20 were identified in 16 neuropeptide genes and their functional significance was evaluated. The product of these genes appeared to affect multiple systems and some were associated with ethnic differences in incidence of common human diseases such as high blood pressure and type 2 diabetes. Significantly, our analysis adds to our knowledge of the genetic variation in continental human populations across all known neuropeptide genes. It also highlights the pleiotropic nature of these neuropeptides, their functional significance in extra neuronal tissues and their association with cardiovascular and metabolic diseases.

## Methods

### Data sets

A list of human neuropeptide genes was manually generated by integrating information from neuropeptide databases, Ensembl, and AmiGO Gene Ontology. Four neuropeptide databases were used for obtaining the gene list and included: StraPep [22], neuropeptides.nl [23], NeuroPedia [24] and NeuroPep [25]. This primary gene list was generated by using the search term “*Homo sapiens*”. The list was further refined by adding more neuropeptide genes using the search term “*Neuropeptide*” in *Homo sapiens* in Ensembl (Ensembl GRCh37.p13) [26] and AmiGO Gene Ontology [27]. In addition, for AmiGO the following GO terms were also used:

- Gene ontology - Molecular function
  - GO:0005184 neuropeptide hormone activity
  - GO:0051428 peptide hormone receptor binding
  - GO:0071855 neuropeptide receptor binding
- Gene ontology - Biological process
  - GO:0007218 neuropeptide signaling pathway

The final list comprised a total of 131 human neuropeptide genes (Fig. 1, Additional file 1: Table S1).

Whole genome sequence data were obtained from a total of 15,164 genomes from gnomAD [21]. This dataset comprises of 6 different populations, Africans (including African Americans), Latino, Ashkenazi Jewish, East Asian, Finnish and Non-Finnish European (Additional file 11: Table S4), which were sequenced between 20 to 30X depth of coverage.

### Genetic diversity

The genetic differences between the African and non-African populations in the gnomAD sequence dataset were characterized using SNPs. The ancestral states of each SNP were determined by the Ensembl Biomart tools [52]. If the ancestral state of the SNP was not provided in Ensembl, a comparison between the allele with the primates using Ensembl multiple primate’s alignment was performed, and the consensus primate allele was used as the ancestral allele for that SNP. Based on the ancestral state, derived allele frequency was tabulated for each SNP and absolute differences of the ΔDAF between African and non-African populations were estimated.

### Functional annotations of selected genes

SNPs were filtered by ΔDAF |(African - non-African)| ≥ 0.20, as this was above the 99^th^ percentile of the distribution. All outlier SNPs were functionally annotated using the VEP tool [53] to determine the most severe consequence for each variant. The primary interest was to see if there were any highly differentiated missense variants or SNPs with high impact consequences. Selected neuropeptide genes in which ΔDAF |(African - non-African)| ≥ 0.20 were further explored with GeneCards [54] database to retrieve information and related function of the selected genes. In addition, genes with these highly differentiated SNPs were also characterized by their presence in human disease databases such as the OMIM [29] and GWAS catalogue [28] to understand the implication of these functional consequences. Furthermore, GTEx portal [30] was also used to explore whether any of these variants affected the level of neuropeptide genes in different tissues.

### Haplotype networks

Median-joining haplotype networks were constructed for selected genomic regions using the NETWORK software (version 5) package [55], to investigate the relationship between the African and non-African haplotypes. Due to the non-availability of individual sequences in the gnomAD dataset, all samples from three representative continental 1000 Genomes Project populations [20], that were whole genome sequenced at low coverage (Mean 7.6X), were used to construct the haplotype networks. For this purpose, we used a total of 620 individuals representing 3 major continental populations. These included 216 Yoruba in Ibadan (YRI), 206 Han Chinese in Beijing (CHB) and 198 Utah Residents (CEPH) with Northern and Western European Ancestry (CEU), representing African, East Asian and European continental populations, respectively. The window sizes of the haplotype networks were selected based on pairwise LD values of r^2^ ≤ 0.2 between the most differentiated and other SNPs in the region (Additional file 12: Table S5). Besides, F_ST_ was also calculated for these highly differentiated SNPs using the 1000 Genomes Project YRI, CHB and CEU samples (Table 1).

## Supplementary information

**Additional file 1 **: **Table S1.** List of 131 neuropeptide genes in human.

**Additional file 2 **: **Table S2.** General information on the variants data for 131 neuropeptide genes.

**Additional file 3 **: **Figure S1.** Distribution of ΔDAF values between Africans and non-Africans.

**Additional file 4 **: **Table S3.** SNPs with absolute differences in derived allele frequencies between African and non-African population equal to or more than 0.20. The data are sorted by differences of derived allele frequency.

**Additional file 5 **: **Figure S2.** Annotating 5163 SNPs consequences using the Ensembl Variant Effect Predictor (VEP) tool.

**Additional file 6 **: **Figure S3.** Median-joining haplotype networks for SNPs listed in Table 1.

**Additional file 7 **: **Figure S4.** Median-joining haplotype networks for selected genomic regions in Africans (YRI) and non-Africans (CEU + CHB). A). Five kb region encompassing *INS rs3842753* (average ΔDAF = 0.097 for all SNPs in the region). B). Another 5 kb region on the same chromosome 11 where ΔDAF = 0.012 between Africans and non-Africans. Both these genomic regions do not have any SNPs with ΔDAF ≥0.20.

**Additional file 8 **: **Figure S5.** Haplotype network of a 14 kb region encompassing *CHGB* in Africans (YRI), East Asians (CHB) and Europeans (CEU).

**Additional file 9 **: **Figure S6.** Haplotype network of a 1 kb region encompassing *IGF2* in Africans (YRI), East Asians (CHB) and Europeans (CEU).

**Additional file 10 **: **Figure S7.** Haplotype network of a 1 kb region encompassing *INS* in Africans (YRI), East Asians (CHB) and Europeans (CEU).

**Additional file 11 **: **Table S4.** Analyzed populations and samples. Populations were split into two categories. Sample size indicates the number of individuals for each population.

**Additional file 12 **: **Table S5.** LD patterns in Africans and non-Africans for highly differentiated SNPs listed in Table 1.

## Data Availability

Not applicable.

## Abbreviations

CEU: Utah Residents (CEPH) with Northern and Western European Ancestry
CHB: Han Chinese in Beijing
DAF: Derived Allele Frequencies
gnomAD: Genome Aggregation Database
GTEx: Genotype-Tissue Expression
GWAS: Genome Wide Association Studies
HAPC: High Altitude Polycythemia
LD: Linkage Disequilibrium
OMIM: Online Mendelian Inheritance in Man
SNP: Single Nucleotide Polymorphisms
T1D: Type 1 Diabetes
T2D: Type 2 Diabetes
VEP: Variant Effect Predictor
YRI: Yoruba in Ibadan

## References

[1] Chang MM, Leeman SE, Niall HD (1971). Amino-acid sequence of substance P. Nat New Biol.

[2] Klavdieva MM (1996). The history of neuropeptides II. Front Neuroendocrinol.

[3] Euler USV, Gaddum JH (1931). An unidentified depressor substance in certain tissue extracts. J Physiol.

[4] Burbach JP (2011). What are neuropeptides?. Methods Mol Biol.

[5] Hökfelt T, Bartfai T, Bloom F (2003). Neuropeptides: opportunities for drug discovery. Lancet Neurol.

[6] Catalani E, De Palma C, Perrotta C, Cervia D (2017). Current evidence for a role of neuropeptides in the regulation of autophagy. Biomed Res Int.

[7] Brain SD, Cox HM (2006). Neuropeptides and their receptors: innovative science providing novel therapeutic targets. Br J Pharmacol.

[8] Hughes J, Woodruff GN (1992). Neuropeptides. Function and clinical applications. Arzneimittelforschung..

[9] Russo AF (2017). Overview of neuropeptides: awakening the senses?. Headache.

[10] Liu L, Zhang Y, Zhang Z, Zhao Y, Fan X, Ma L, et al. Associations of high altitude polycythemia with polymorphisms in EPHA2 and AGT in Chinese Han and Tibetan populations. Oncotarget. 2017;8(32):53234–43. 10.18632/oncotarget.18384 PMC5581106 28881807

[11] Cannone V, Boerrigter G, Costello-Boerrigter LC, Cataliotti A, Bailey KR, Lahr B (2010). Association of NPPA rs5065 genetic variant with increased cardiovascular risk in the General USA population. J Card Fail.

[12] Srivastava A, Mittal B, Prakash J, Narain VS, Natu SM, Srivastava N (2014). Evaluation of MC4R [rs17782313, rs17700633], AGRP [rs3412352] and POMC [rs1042571] polymorphisms with obesity in northern India. Oman Med J.

[13] Kormos V, Gaszner B (2013). Role of neuropeptides in anxiety, stress, and depression: from animals to humans. Neuropeptides..

[14] Scholzen T, Armstrong CA, Bunnett NW, Luger TA, Olerud JE, Ansel JC (1998). Neuropeptides in the skin: interactions between the neuroendocrine and the skin immune systems. Exp Dermatol.

[15] van den Pol AN (2012). Neuropeptide transmission in brain circuits. Neuron..

[16] Iijima Y, Inada T, Ohtsuki T, Senoo H, Nakatani M, Arinami T (2004). Association between chromogranin b gene polymorphisms and schizophrenia in the Japanese population. Biol Psychiatry.

[17] Lappalainen J, Kranzler HR, Malison R, Price LH, Van Dyck C, Rosenheck RA (2002). A functional neuropeptide Y Leu7Pro polymorphism associated with alcohol dependence in a large population sample from the United States. Arch Gen Psychiatry.

[18] Zhu G, Pollak L, Mottagui-Tabar S, Wahlestedt C, Taubman J, Virkkunen M (2003). NPY Leu7Pro and alcohol dependence in Finnish and Swedish populations. Alcohol Clin Exp Res.

[19] Shin JG, Kim JH, Park CS, Kim BJ, Kim JW, Choi IG (2017). Gender-specific associations between CHGB genetic variants and schizophrenia in a Korean population. Yonsei Med J.

[20] The 1000 Genomes Project Consortium (2015). A global reference for human genetic variation. Nat.

[21] Karczewski KJ, Francioli LC, Tiao G, Cummings BB, Alföldi J, Wang Q, et al. Variation across 141,456 human exomes and genomes reveals the spectrum of loss-of-function intolerance across human protein-coding genes. BioRxiv. 2019:531210..

[22] Wang J, Yin T, Xiao X, He D, Xue Z, Jiang X, Wang Y. StraPep: a structure database of bioactive peptides. Database. 2018;2018:bay038. 10.1093/database/bay038 PMC5905355 29688386

[23] Burbach JP (2010). Neuropeptides from concept to online database www. Neuropeptides. Nl. Eur J Pharmacol.

[24] Kim Y, Bark S, Hook V, Bandeira N (2011). NeuroPedia: neuropeptide database and spectral library. Bioinformatics..

[25] Wang Y, Wang M, Yin S, Jang R, Wang J, Xue Z, Xu T. NeuroPep: a comprehensive resource of neuropeptides. Database. 2015;2015:bav038. 10.1093/database/bav038 PMC4414954 25931458

[26] Howe KL, Contreras-Moreira B, De Silva N, Maslen G, Akanni W, Allen J, Alvarez-Jarreta J, Barba M, Bolser DM, Cambell L, Carbajo M. Ensembl genomes 2020—enabling non-vertebrate genomic research. Nucleic Acids Res. 2020;48(D1):D689–95. 10.1093/nar/gkz890 PMC6943047 31598706

[27] Carbon S, Ireland A, Mungall CJ, Shu S, Marshall B, Lewis S (2008). AmiGO hub, web presence working group. AmiGO: online access to ontology and annotation data. Bioinformatics.

[28] Buniello A, MacArthur JA, Cerezo M, Harris LW, Hayhurst J, Malangone C, McMahon A, Morales J, Mountjoy E, Sollis E, Suveges D (2018). The NHGRI-EBI GWAS catalog of published genome-wide association studies, targeted arrays and summary statistics 2019. Nucleic Acids Res.

[29] Hamosh A, Scott AF, Amberger JS, Bocchini CA, McKusick VA (2005). Online Mendelian inheritance in man (OMIM), a knowledgebase of human genes and genetic disorders. Nucleic Acids Res.

[30] GTEx Consortium (2015). The genotype-tissue expression (GTEx) pilot analysis: multitissue gene regulation in humans. Science.

[31] Cannone V, Huntley BK, Olson TM, Heublein DM, Scott CG, Bailey KR (2013). Atrial natriuretic peptide genetic variant rs5065 and risk for cardiovascular disease in the general community: a 9-year follow-up study. Hypertension.

[32] Barbato E, Bartunek J, Mangiacapra F, Sciarretta S, Stanzione R, Delrue L (2012). Influence of rs5065 atrial natriuretic peptide gene variant on coronary artery disease. J Am Coll Cardiol.

[33] Zhang K, Rao F, Rana BK, Gayen JR, Calegari F, King A (2009). Autonomic function in hypertension; role of genetic variation at the catecholamine storage vesicle protein chromogranin B. Circ Cardiovasc Genet.

[34] Fabregat M, Fernandez M, Javiel G, Vitarella G, Mimbacas A (2015). The genetic profile from HLA and non-HLA loci allows identification of atypical type 2 diabetes patients. J Diabetes Res.

[35] Mueller PW, Rogus JJ, Cleary PA, Zhao Y, Smiles AM, Steffes MW (2006). Genetics of kidneys in diabetes (GoKinD) study: a genetics collection available for identifying genetic susceptibility factors for diabetic nephropathy in type 1 diabetes. J Am Soc Nephrol.

[36] Gu T, Horová E, Möllsten A, Seman NA, Falhammar H, Prázny M (2012). IGF2BP2 and IGF2 genetic effects in diabetes and diabetic nephropathy. J Diabetes Complicat.

[37] Sergeeva IA, Hooijkaas IB, Van Der Made I, Jong WM, Creemers EE, Christoffels VM (2014). A transgenic mouse model for the simultaneous monitoring of ANF and BNP gene activity during heart development and disease. Cardiovasc Res.

[38] Simonetti G, Mohaupt M (2007). Calcium and blood pressure. Ther Umsch.

[39] Yadav GP, Zheng H, Yang Q, Douma LG, Bloom LB, Jiang QX (2018). Secretory granule protein chromogranin B (CHGB) forms an anion channel in membranes. Life Sci Alliance.

[40] Douglas WW, Rubin RP (1963). The mechanism of catecholamine release from the adrenal medulla and the role of calcium in stimulus-secretion coupling. J Physiol.

[41] Ayada C, Toru Ü, Korkut Y (2015). Nesfatin-1 and its effects on different systems. Hippokratia.

[42] Goebel-Stengel M, Stengel A (2016). Role of Brain NUCB2/nesfatin-1 in the stress-induced modulation of gastrointestinal functions. Curr Neuropharmacol.

[43] Ozcan M, Gok ZB, Kacar E, Serhatlioglu I, Kelestimur H (2016). Nesfatin-1 increases intracellular calcium concentration by protein kinase C activation in cultured rat dorsal root ganglion neurons. Neurosci Lett.

[44] Tragante V, Barnes MR, Ganesh SK, Lanktree MB, Guo W, Franceschini N (2014). Gene-centric meta-analysis in 87,736 individuals of European ancestry identifies multiple blood-pressure-related loci. Am J Hum Genet.

[45] Li Y. Detecting association of common and rare variants with complex diseases. PhD [dissertation]. Ohio: Case Western Reserve University; 2010.

[46] Yako YY, Balti EV, Matsha TE, Dzudie A, Kruger D, Sobngwi E (2018). Genetic factors contributing to hypertension in African-based populations: a systematic review and meta-analysis. J Clin Hypertens (Greenwich).

[47] Cheng CY, Reich D, Haiman CA, Tandon A, Patterson N, Selvin E (2012). African ancestry and its correlation to type 2 diabetes in African Americans: a genetic admixture analysis in three U.S. population cohorts. PLoS One.

[48] Ojha A, Ojha U, Mohammed R, Chandrashekar A, Ojha H (2019). Current perspective on the role of insulin and glucagon in the pathogenesis and treatment of type 2 diabetes mellitus. Clin Pharmacol.

[49] Chang CL, Cai JJ, Lo C, Amigo J, Park JI, Hsu SY (2011). Adaptive selection of an incretin gene in Eurasian populations. Genome Res.

[50] Wang C, Wang Y, Hu W (2017). Association of the polymorphism in NUCB2 gene and the risk of type 2 diabetes. Diabetol Metab Syndr.

[51] Obermüller S, Calegari F, King A, Lindqvist A, Lundquist I, Salehi A (2010). Defective secretion of islet hormones in chromogranin-B deficient mice. PLoS One.

[52] Kinsella RJ, Kähäri A, Haider S, Zamora J, Proctor G, Spudich G (2011). Ensembl BioMarts: a hub for data retrieval across taxonomic space. Database..

[53] McLaren W, Gil L, Hunt SE, Riat HS, Ritchie GR, Thormann A (2016). The Ensembl variant effect predictor. Genome Biol.

[54] Safran M, Dalah I, Alexander J, Rosen N, Iny Stein T, Shmoish M, Nativ N, Bahir I, Doniger T, Krug H, Sirota-Madi A (2010). GeneCards version 3: the human gene integrator. Database..

[55] Bandelt HJ, Forster P, Röhl A (1999). Median-joining networks for inferring intraspecific phylogenies. Mol Biol Evol.

