# Supplementary figures and images for: Selected neuropeptide genes show genetic differentiation between Africans and non-Africans
Source: BMC Genet. 2020 Mar 14;21:31. doi: 10.1186/s12863-020-0835-8 (PMC7071772; doi:10.1186/s12863-020-0835-8)

Figure S1. Distribution of  $\Delta DAF$  values between Africans and non-Africans.

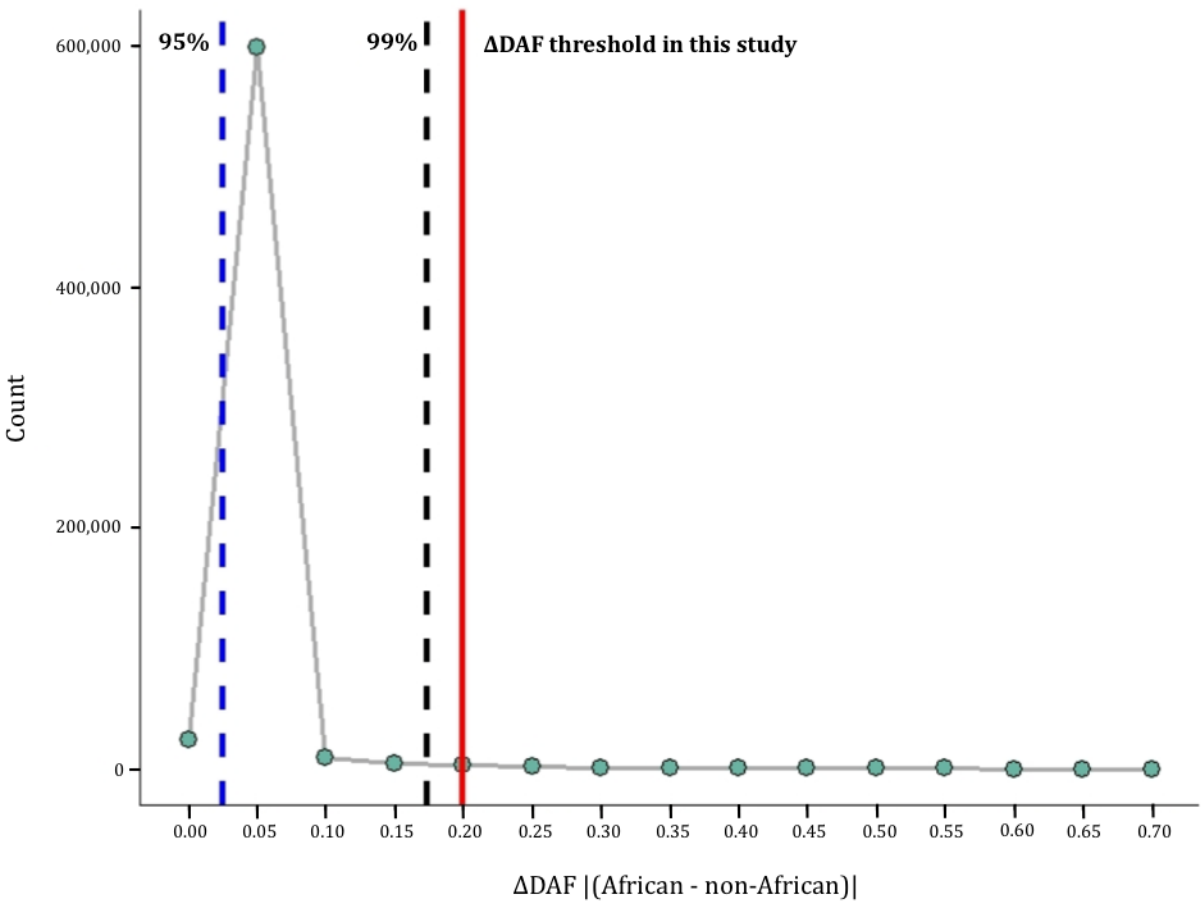

Supplement: Supplementary file 3 — Additional file 3 : Figure S1. Distribution of ΔDAF values between Africans and non-Africans. [file 12863_2020_835_MOESM3_ESM.pdf]

Figure S2. Annotating 5,163 SNPs consequences using the Ensembl Variant Effect Predictor (VEP) tool.

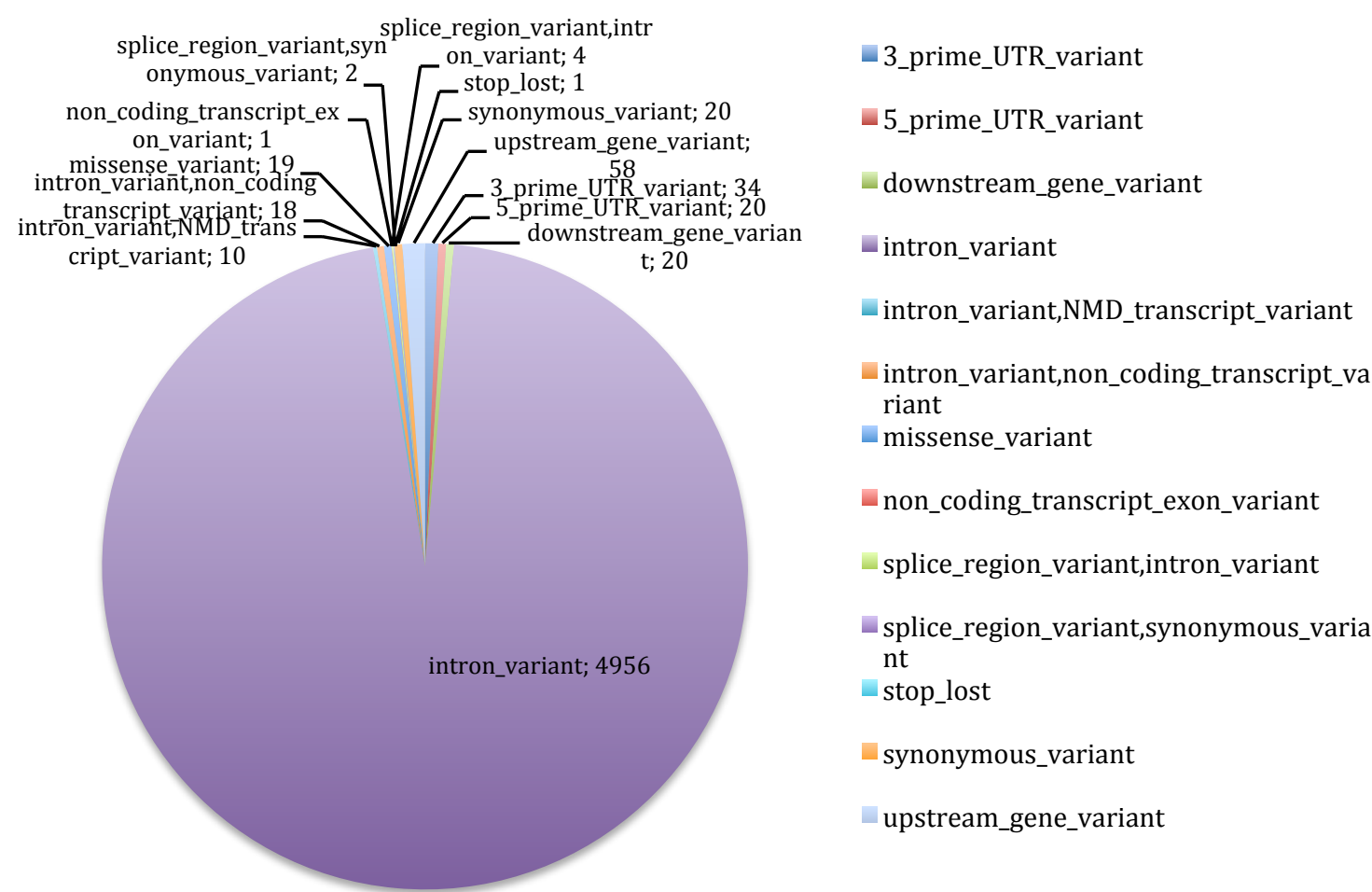

Supplement: Supplementary file 5 — Additional file 5 : Figure S2. Annotating 5163 SNPs consequences using the Ensembl Variant Effect Predictor (VEP) tool. [file 12863_2020_835_MOESM5_ESM.pdf]
