# Supplementary material for: Selected neuropeptide genes show genetic differentiation between Africans and non-Africans
Source: BMC Genet. 2020 Mar 14;21:31. doi: 10.1186/s12863-020-0835-8 (PMC7071772; doi:10.1186/s12863-020-0835-8)

Figure S3. Median-joining haplotype networks for SNPs listed in Table 1.

a. *QRFP*, 9:133768815-133769225, 411 bases

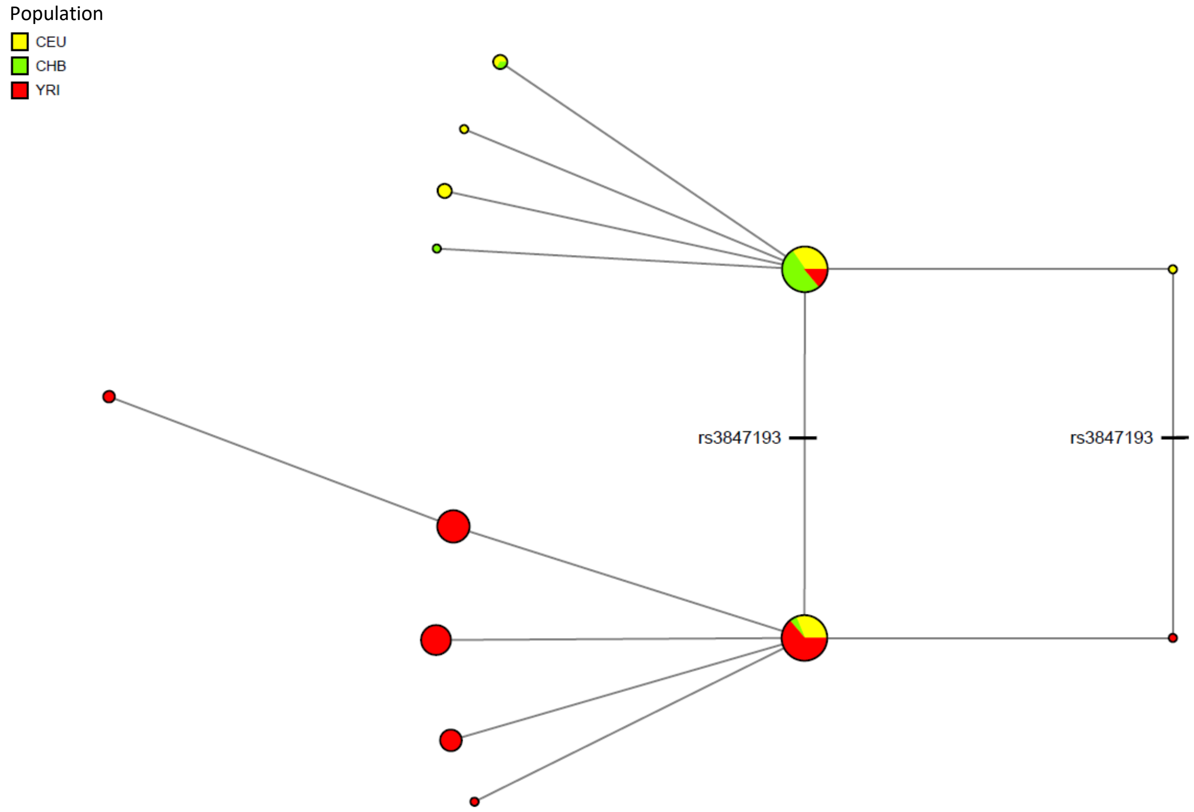

b. *GIP*, 17:47038132-47040131, 2000 bases

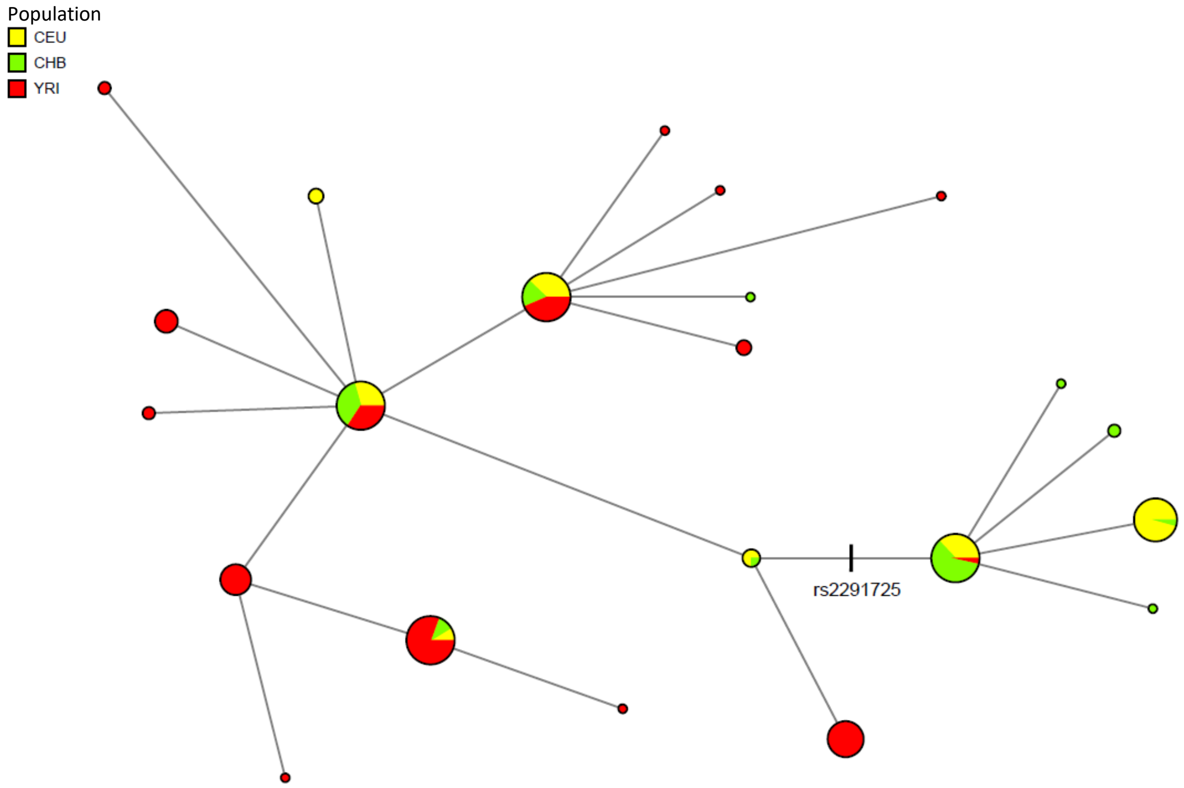

c. *AGT*, 1:230844794-230846793, 2000 bases

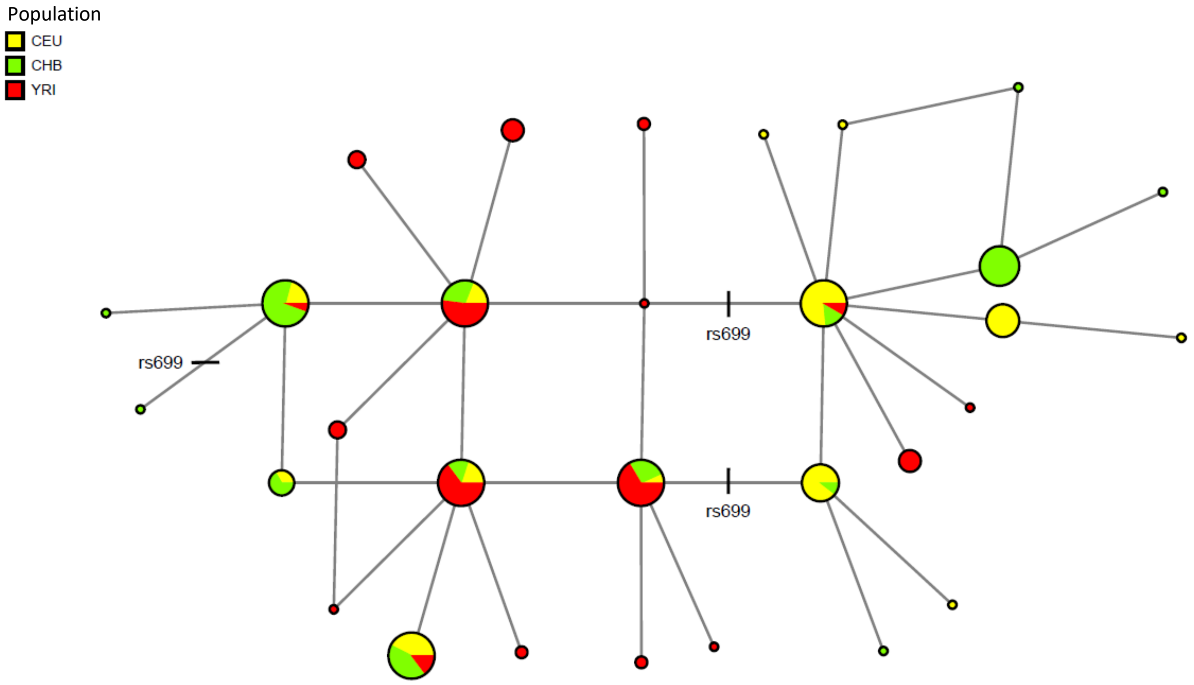

d. *UTS2B*, 3:190998917-191000916, 2000 bases

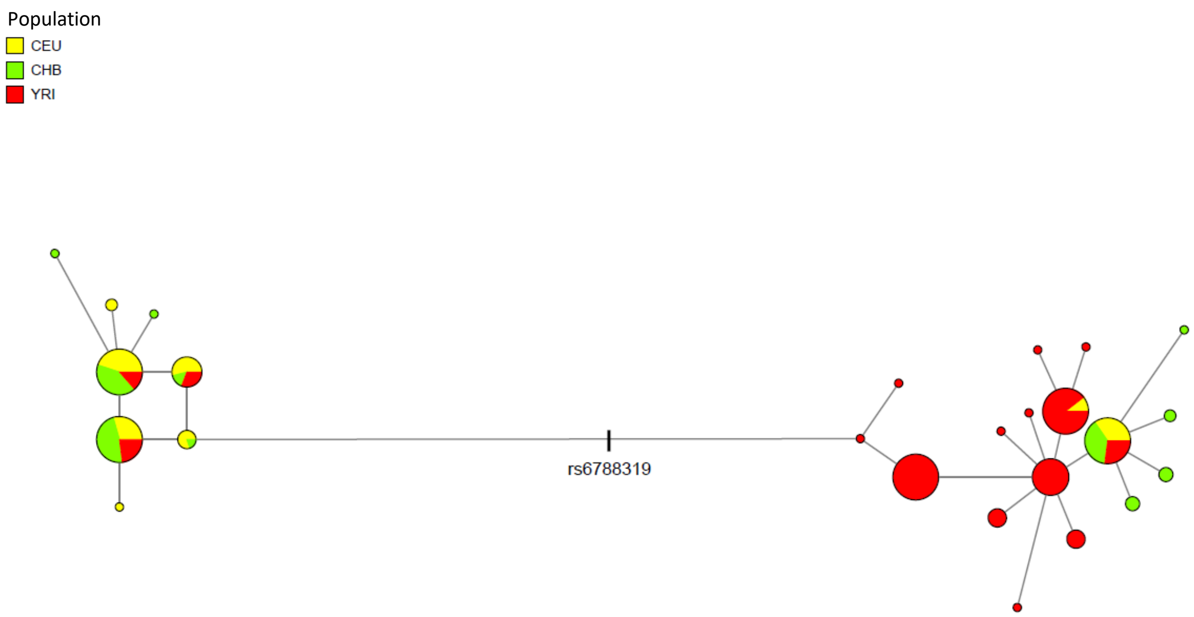

e. *NPW*, 16:2069568-2071567, 2000 bases

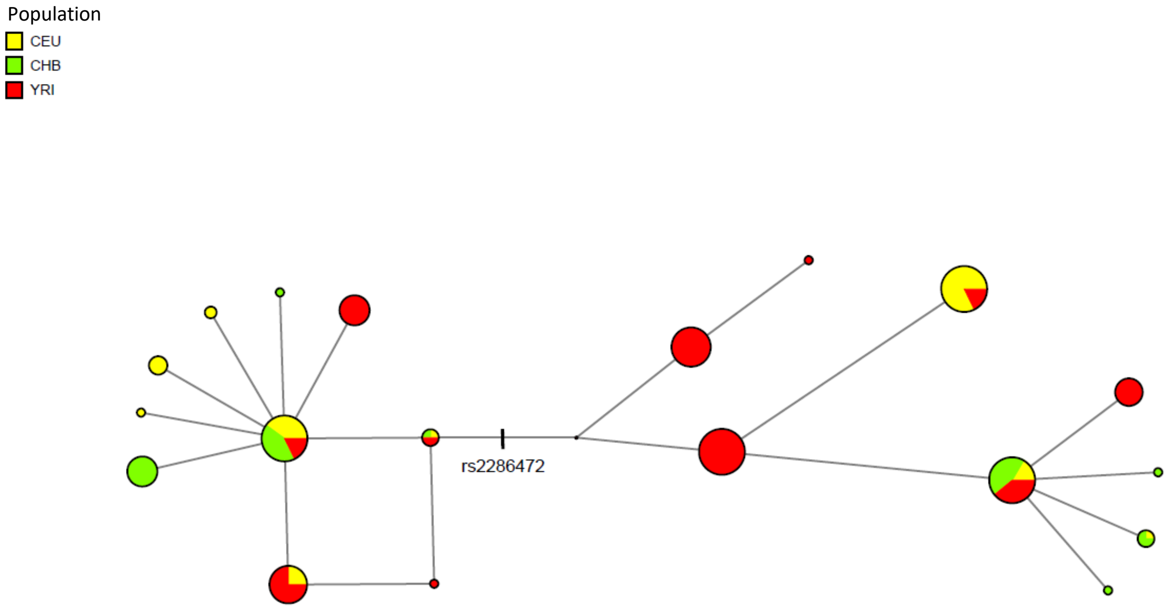

f. *SERPINA11*, 14:94911896-94913895, 2000 bases

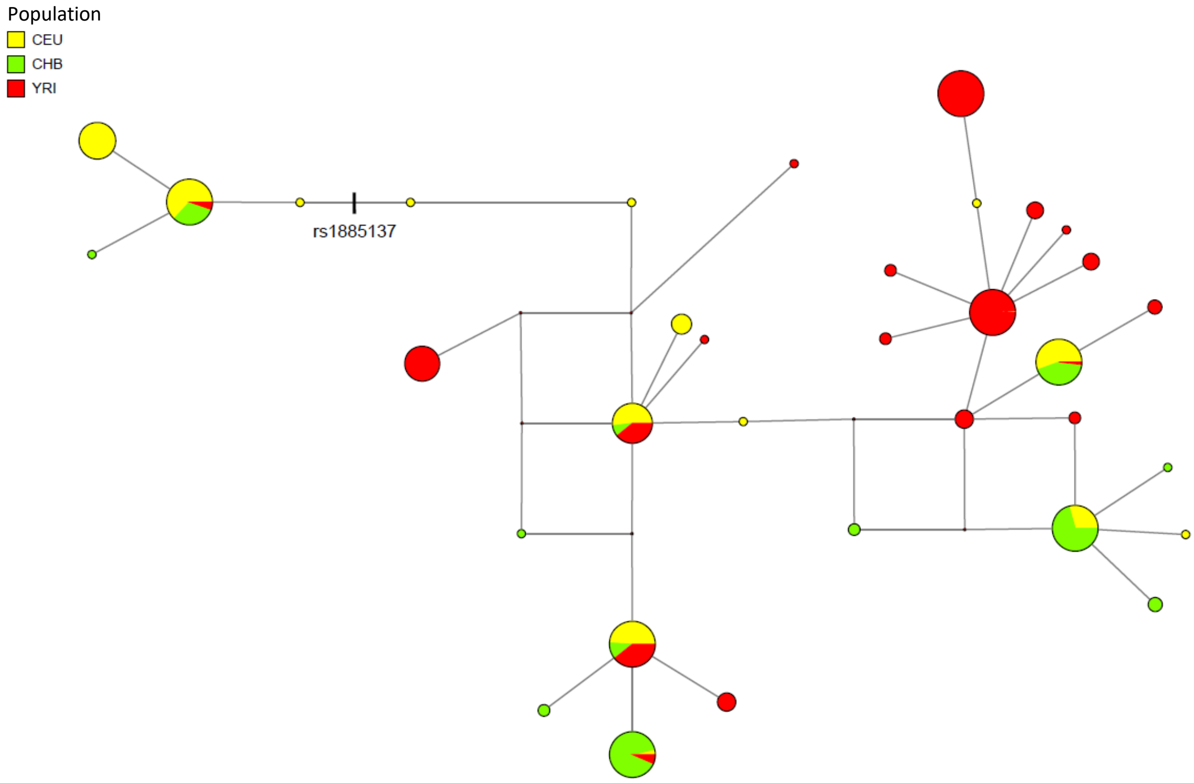

g. *GNAS*, 20:57477807-57479806, 2000 bases

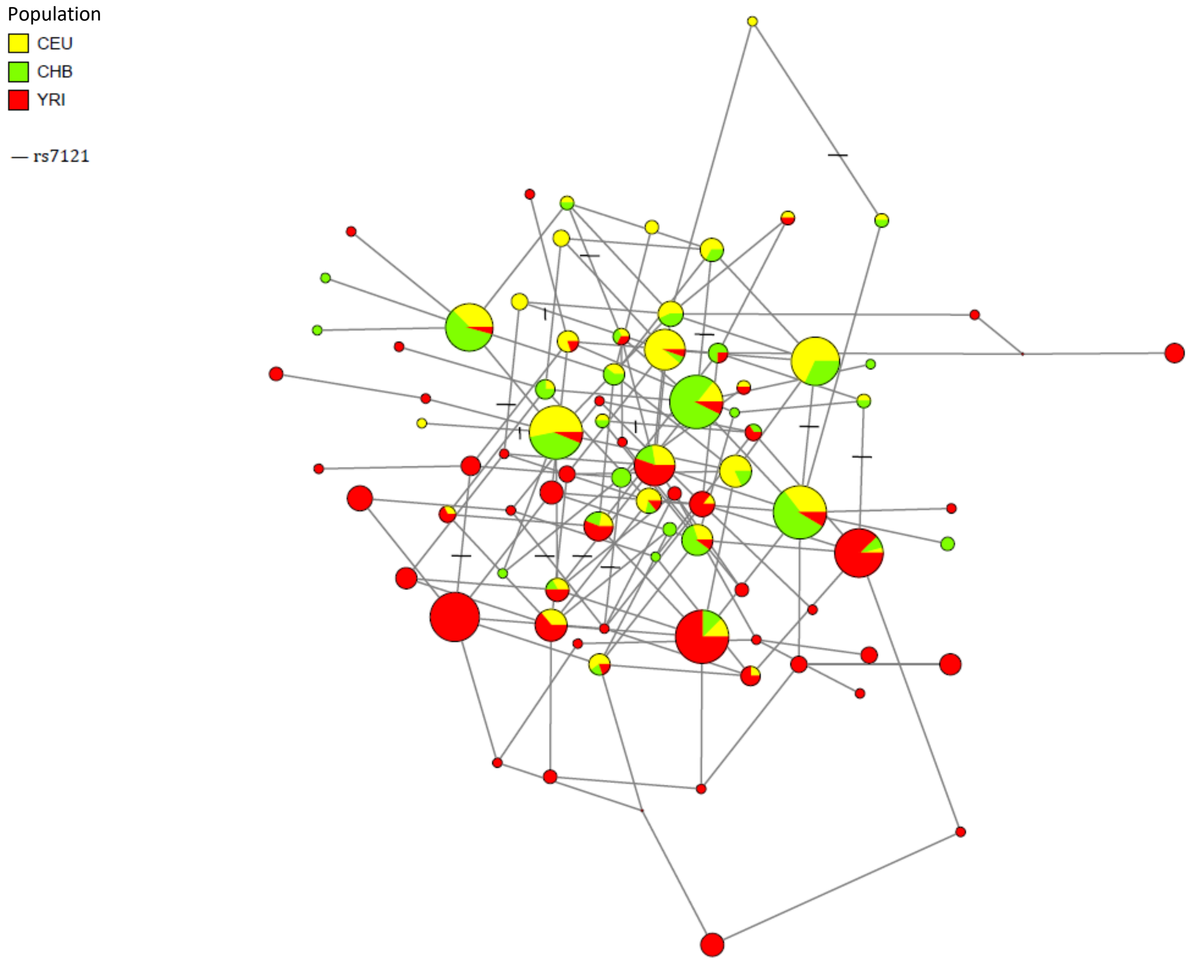

h. *NUCB2*, 11:17350683-17352682, 2000 bases

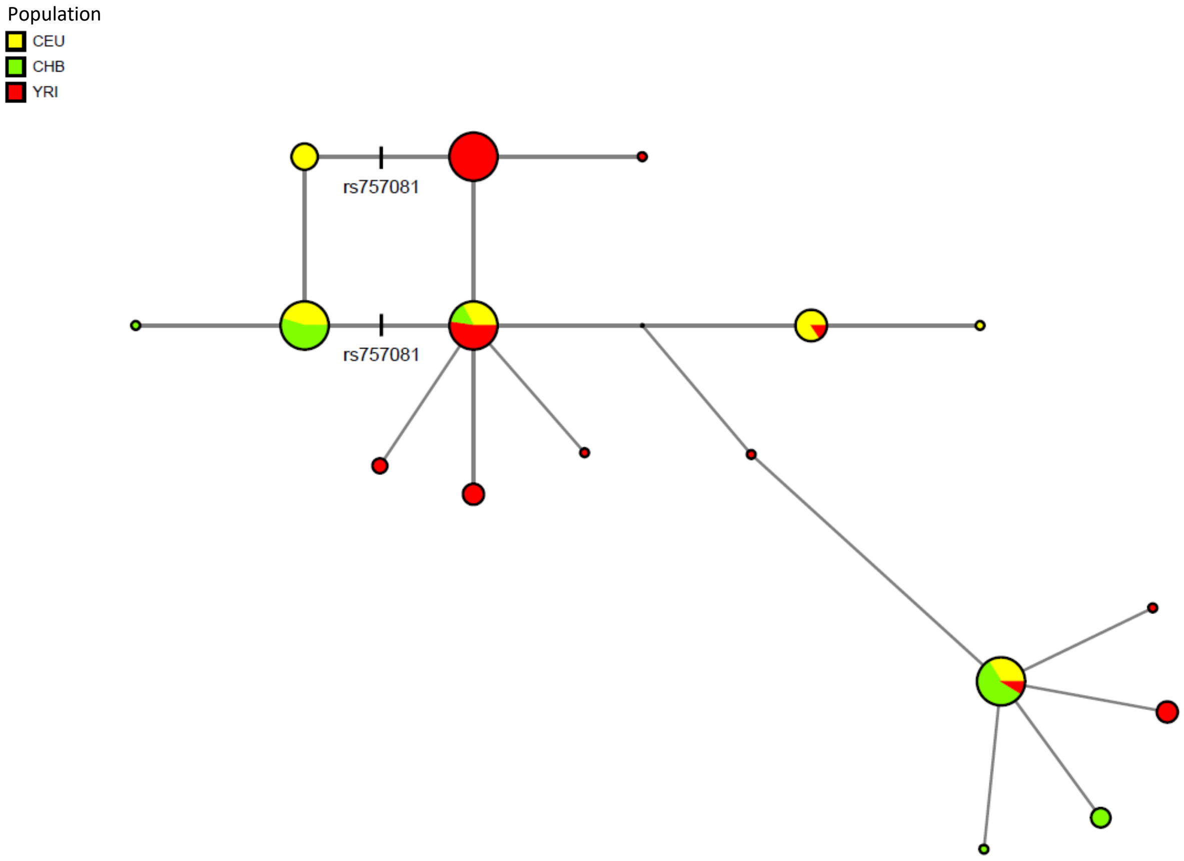

i. *NPS*, 10:129346785-129348784, 2000 bases

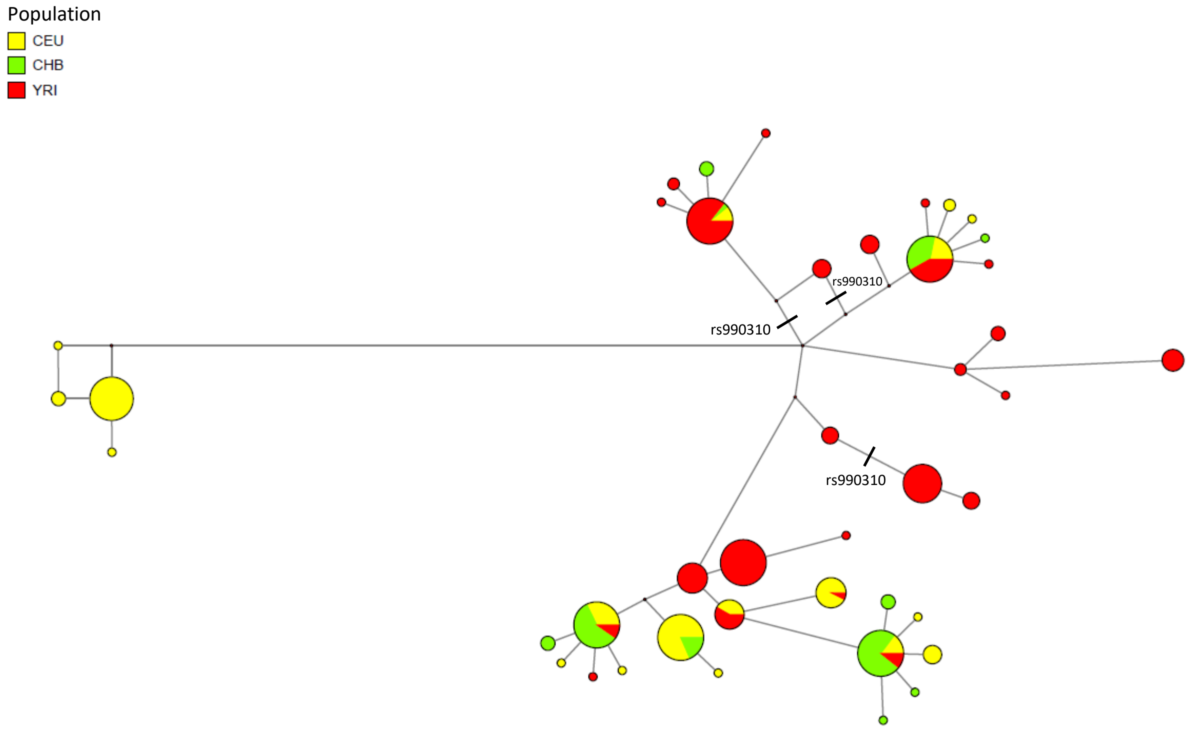

j. *GNAO1*, 16:56376748-56378747, 2000 bases

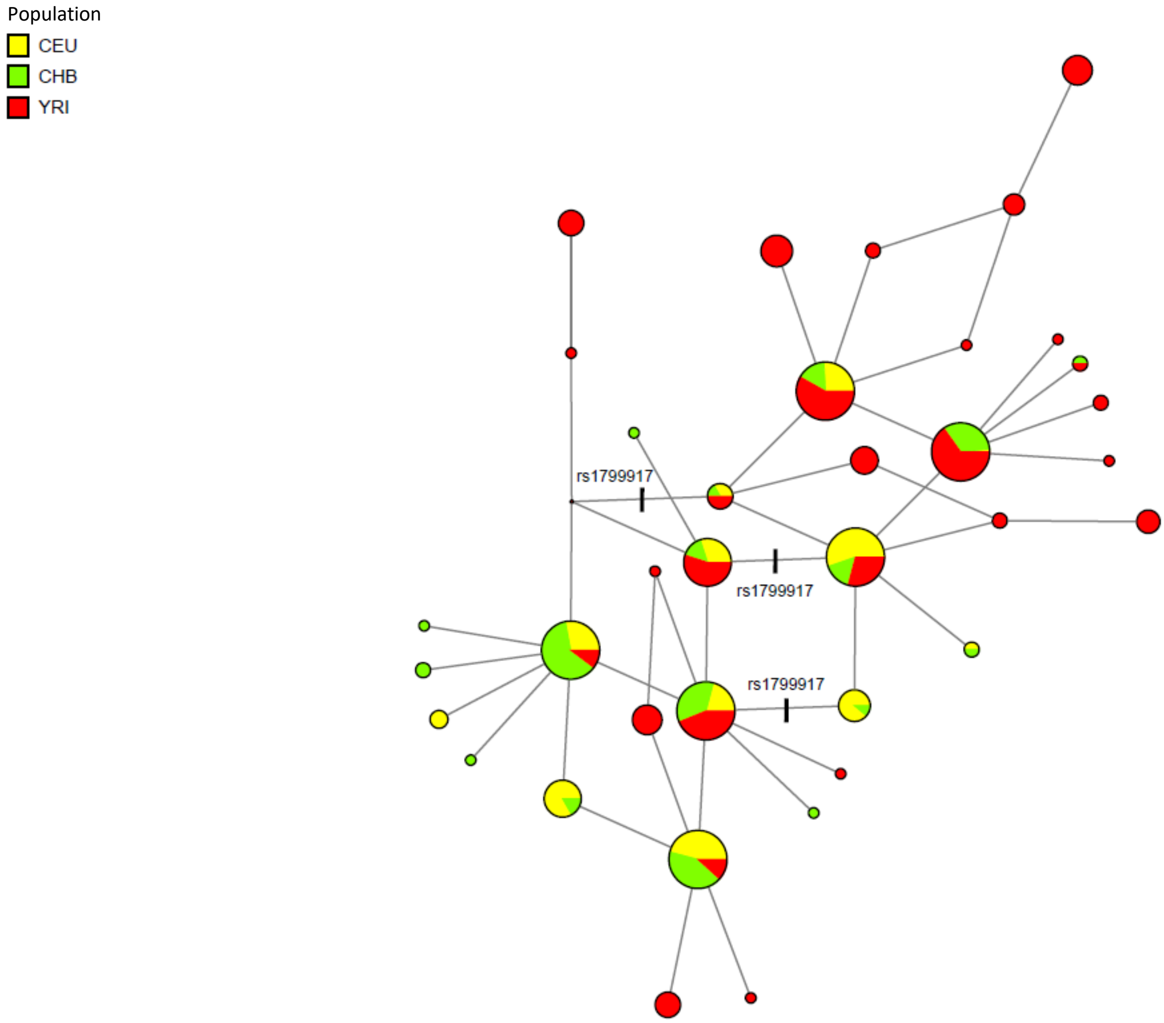

k. *AGRP*, 16:67516474-67517716, 1243 bases

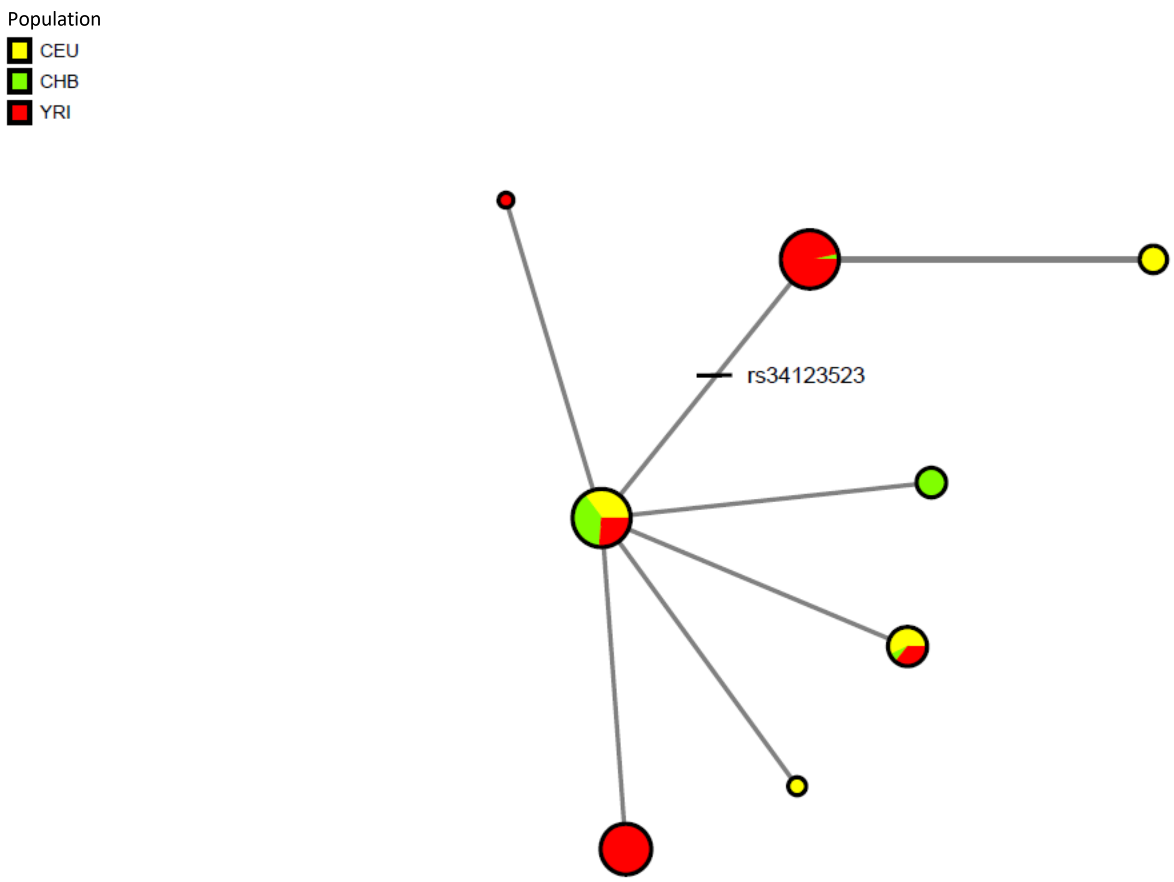

I. *PYY*, 17:42029531-42031530, 2000 bases

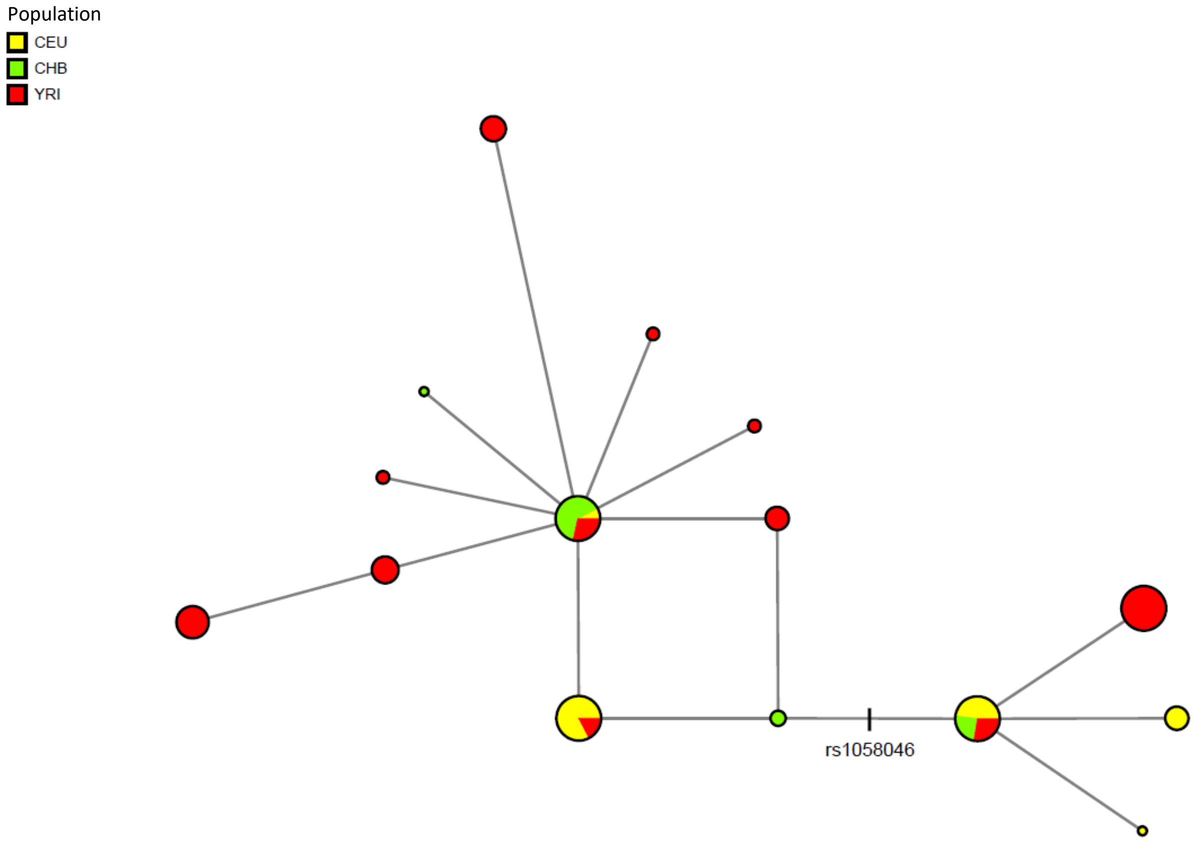

Supplement: Supplementary file 6 — Additional file 6 : Figure S3. Median-joining haplotype networks for SNPs listed in Table 1. [file 12863_2020_835_MOESM6_ESM.pdf]
