# Supplementary material for: Selected neuropeptide genes show genetic differentiation between Africans and non-Africans
Source: BMC Genet. 2020 Mar 14;21:31. doi: 10.1186/s12863-020-0835-8 (PMC7071772; doi:10.1186/s12863-020-0835-8)

Figure S4. Median-joining haplotype networks for selected genomic regions in Africans (YRI) and non-Africans (CEU + CHB).

a. 5 kb region encompassing *INS* rs3842753 (average ΔDAF = 0.097 for all SNPs in the region).

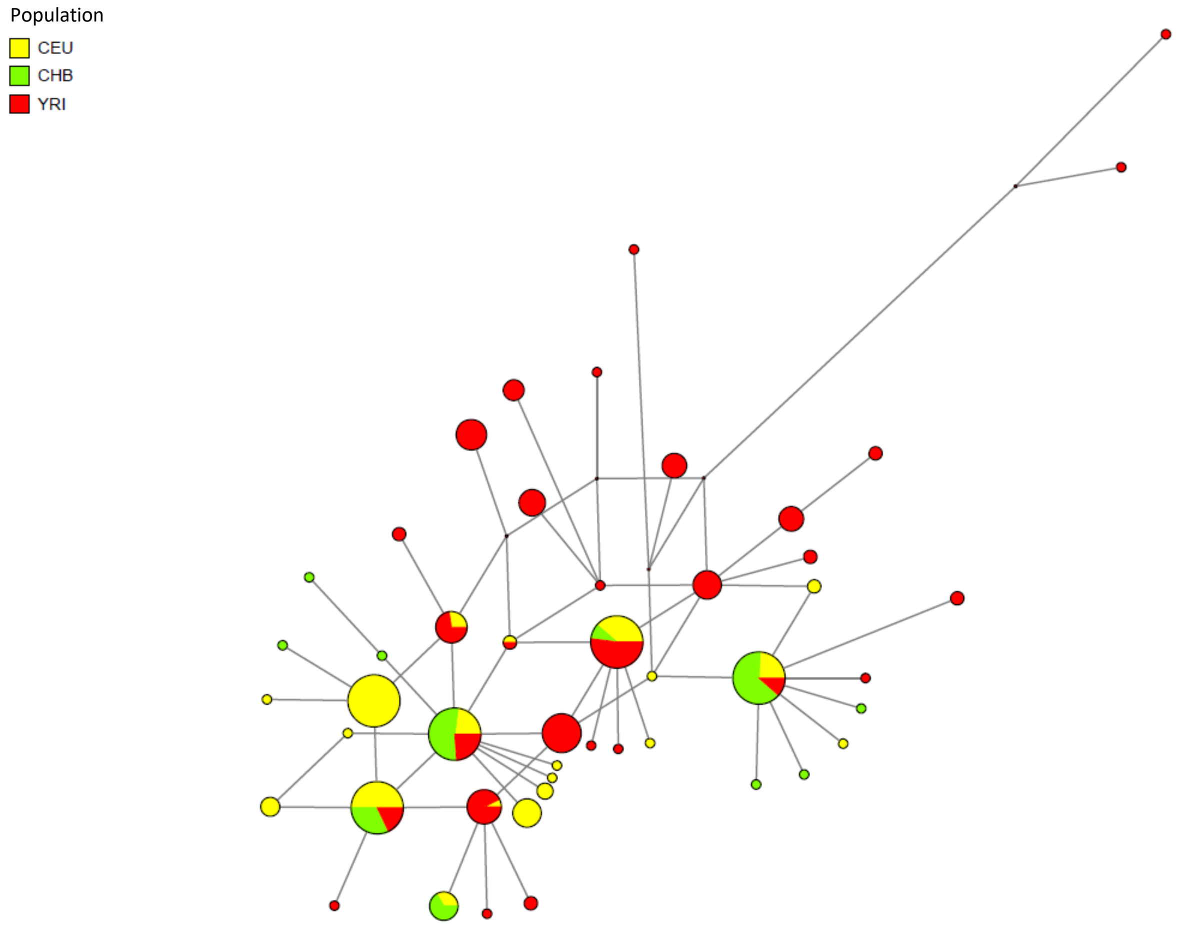

b. Another 5 kb region on the same chromosome 11 where ΔDAF = 0.012 between Africans and non-Africans. Both these genomic regions do not have any SNPs with ΔDAF ≥ 0.20.

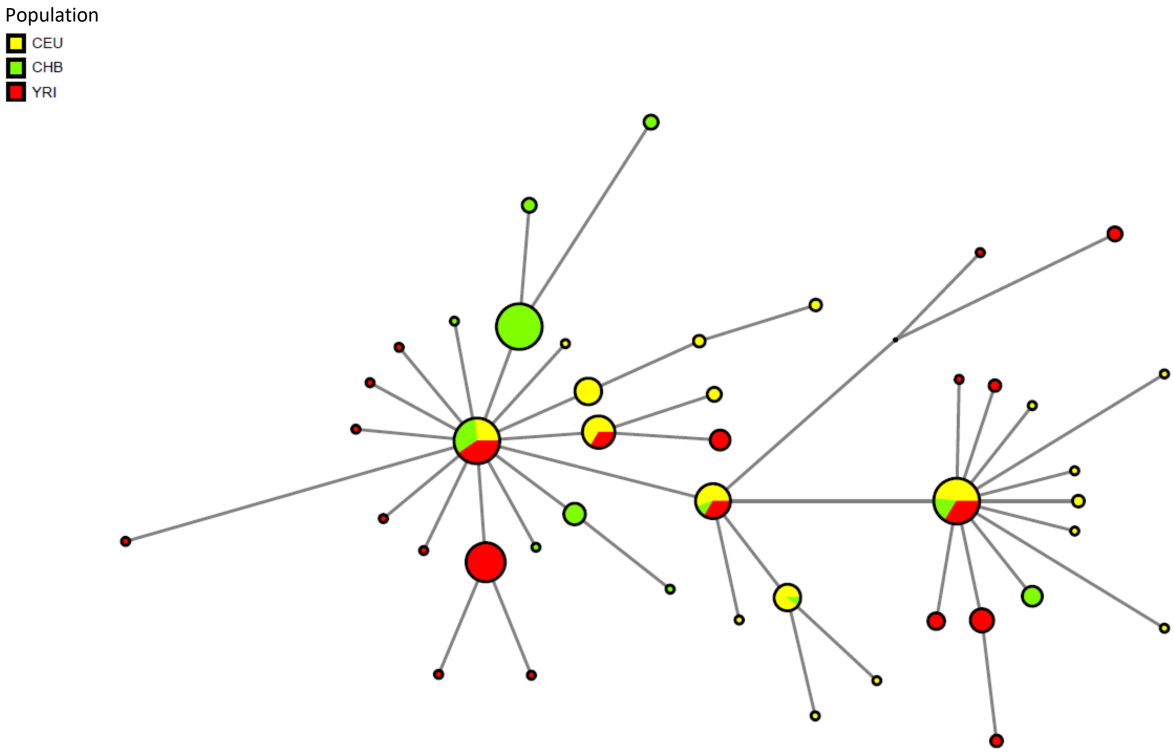

Supplement: Supplementary file 7 — Additional file 7 : Figure S4. Median-joining haplotype networks for selected genomic regions in Africans (YRI) and non-Africans (CEU + CHB). A). Five kb region encompassing INS rs3842753 (average ΔDAF = 0.097 for all SNPs in the region). B). Another 5 kb region on the same chromosome 11 where ΔDAF = 0.012 between Africans and non-Africans. Both these genomic regions do not have any SNPs with ΔDAF ≥0.20. [file 12863_2020_835_MOESM7_ESM.pdf]
