# Supplementary material for: Selected neuropeptide genes show genetic differentiation between Africans and non-Africans
Source: BMC Genet. 2020 Mar 14;21:31. doi: 10.1186/s12863-020-0835-8 (PMC7071772; doi:10.1186/s12863-020-0835-8)

Figure S5. Haplotype network of a 14 kb region encompassing *CHGB* in Africans (YRI), East Asians (CHB) and Europeans (CEU).

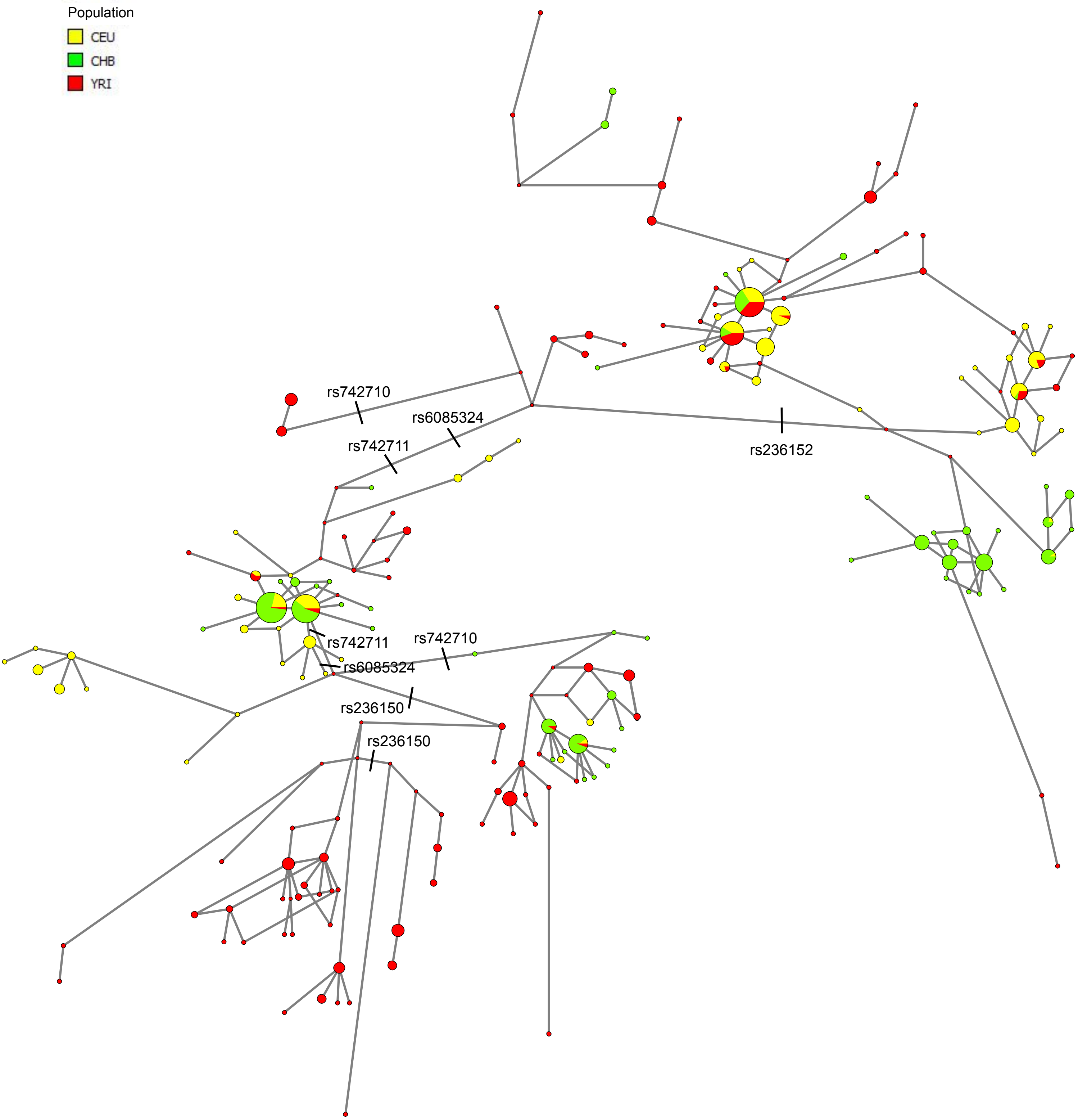

Supplement: Supplementary file 8 — Additional file 8 : Figure S5. Haplotype network of a 14 kb region encompassing CHGB in Africans (YRI), East Asians (CHB) and Europeans (CEU). [file 12863_2020_835_MOESM8_ESM.pdf]
