# Supplementary material for: Selected neuropeptide genes show genetic differentiation between Africans and non-Africans
Source: BMC Genet. 2020 Mar 14;21:31. doi: 10.1186/s12863-020-0835-8 (PMC7071772; doi:10.1186/s12863-020-0835-8)

Figure S6. Haplotype network of a 1 kb region encompassing *IGF2* in Africans (YRI), East Asians (CHB) and Europeans (CEU).

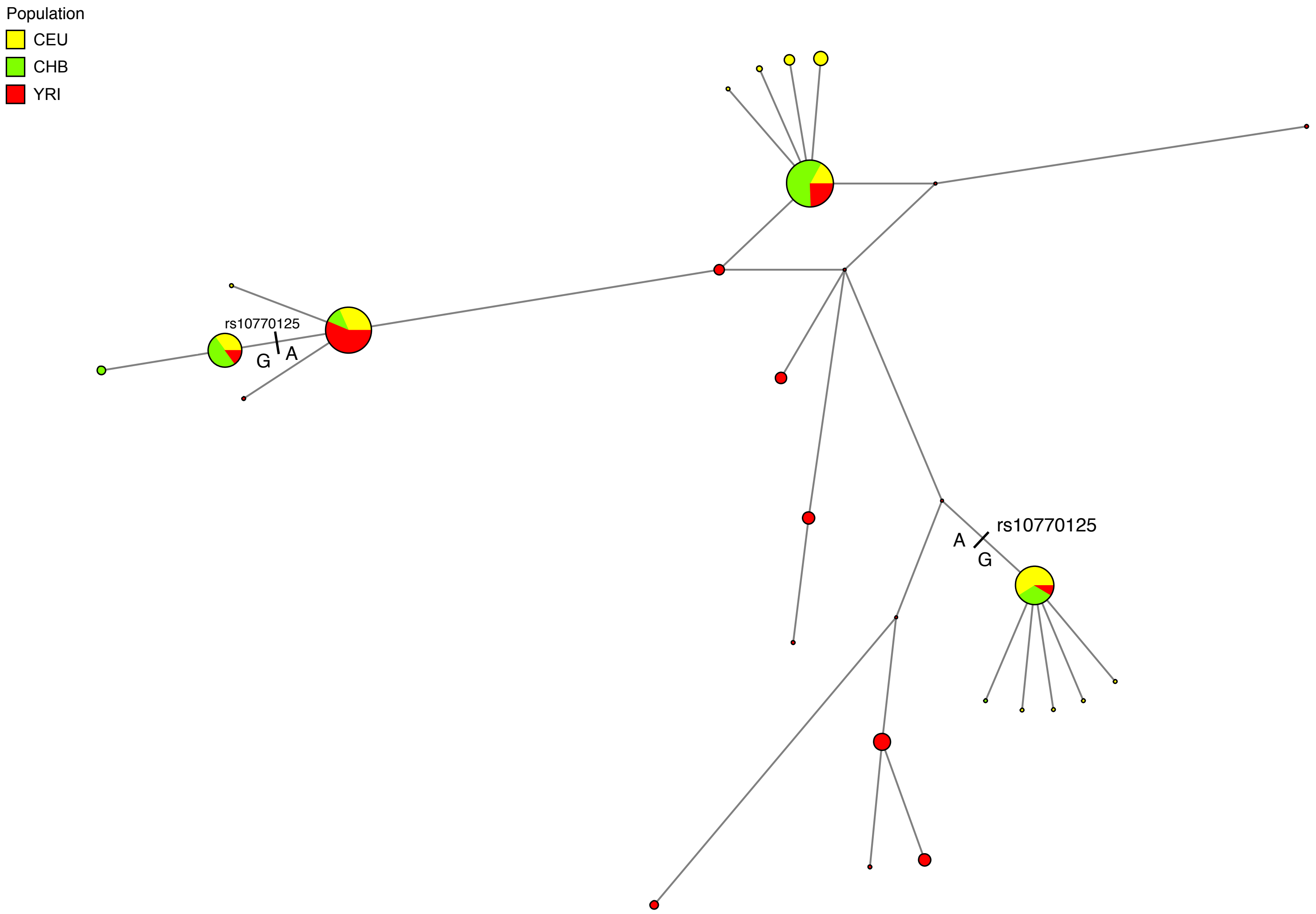

Supplement: Supplementary file 9 — Additional file 9 : Figure S6. Haplotype network of a 1 kb region encompassing IGF2 in Africans (YRI), East Asians (CHB) and Europeans (CEU). [file 12863_2020_835_MOESM9_ESM.pdf]
