# Supplementary material for: Selected neuropeptide genes show genetic differentiation between Africans and non-Africans
Source: BMC Genet. 2020 Mar 14;21:31. doi: 10.1186/s12863-020-0835-8 (PMC7071772; doi:10.1186/s12863-020-0835-8)

Figure S7. Haplotype network of a 1 kb region encompassing *INS* in Africans (YRI), East Asians (CHB) and Europeans (CEU).

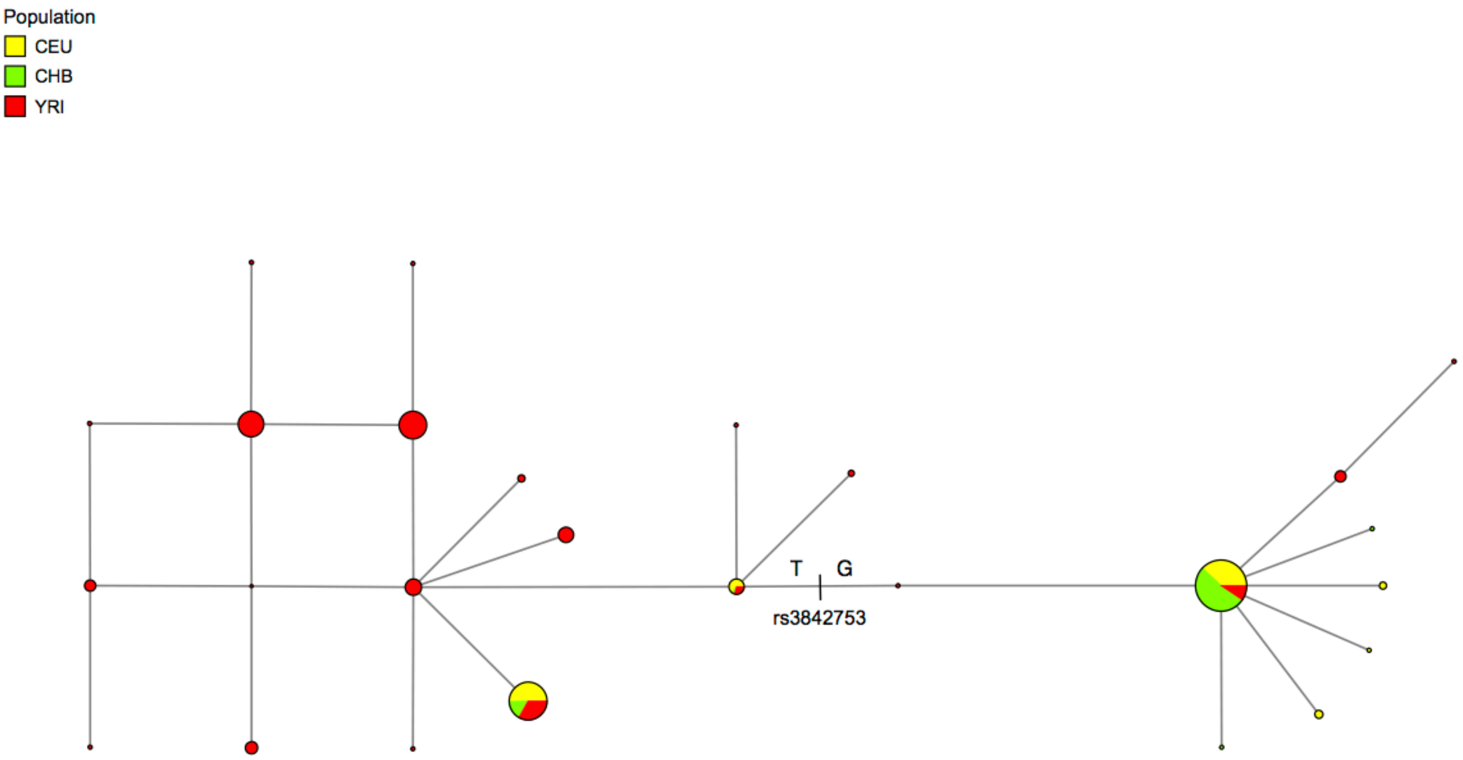

Supplement: Supplementary file 10 — Additional file 10 : Figure S7. Haplotype network of a 1 kb region encompassing INS in Africans (YRI), East Asians (CHB) and Europeans (CEU). [file 12863_2020_835_MOESM10_ESM.pdf]
